# Dynamics of stochastic-constrained particles

**DOI:** 10.1038/s41598-023-29940-y

**Published:** 2023-02-16

**Authors:** Tao Guo

**Affiliations:** Center for Drug Delivery System, Shanghai Institute of Materia Medica, Chinese Academy of Sciences, 501 Haike Road, Shanghai, 201210 China

**Keywords:** Galaxies and clusters, Applied mathematics, Fluid dynamics

## Abstract

Prior studies have focused on the overall behavior of randomly moving particle swarms. However, the characteristics of the stochastic-constrained particles that form ubiquitously within these swarms remain oblivious. This study demonstrates a generalized diffusion equation for stochastic-constrained particles that considers the velocity and location aggregation effects observed from their parent particle swarm (i.e., a completely random particle swarm). This equation can be approximated as the form of Schrödinger equation in the microcosmic case (low relative density) and describe the dynamics of the total mass distribution in the macrocosmic case (high relative density). The predicted density distribution of the particle swarm in the stable aggregation state is consistent with the total mass distribution of massive, relaxed galaxy clusters (at least in the range of $r<r_{\textrm{s}}$), preventing cuspy problems in the empirical Navarro–Frenk–White profile. This study opens a window to observe the dynamics of stochastic-constrained particles from a third perspective, from which the aggregation effect of particles without gravitation can be saw.

## Introduction

**Figure 1:**
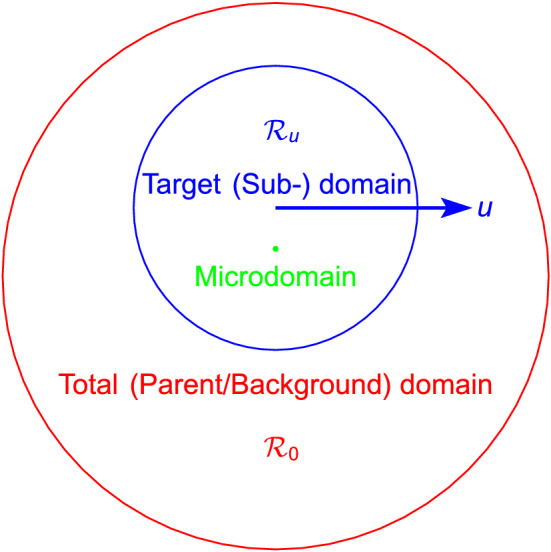
Relationship between the Total (Parent/Background) Domain (Red), Target (Sub-) Domain (Blue) and Microdomain (Green).

The dynamics of randomly moving particles have been extensively studied in the past^1–5^. However, these studies have been based on the cases where the means (velocity and density) of the particles in the target (sub-) domain are identical to those in the total (parent) domain (Fig. 1), or the particle swarm in the sub- and parent domains are indistinguishable. There are certain stochastic-constrained sub-particle swarms with minuscule probabilities in the particle swarm that are formed by the randomly moving particles. For example, during a certain period, the sub-particle swarm (${\mathcal {R}}_u$) with a constant velocity relative to the parent particle swarm^6^ belongs to this category (Fig. 1). These special sub-particle swarms are accidental phenomena for the particles in the parent domain, but for the observers near these sub-particle swarms, they are determined “gifts” from nature (survivor bias). These cases are also the more common existences we see and are meaningful to human beings (if the whole universe is considered as a composition of minute particles, a galaxy in a galaxy cluster, the Solar System in the Milky Way, and the Earth in the Solar System are similar to this type of phenomenon). Therefore, it is extremely necessary to study the particle swarms in common but special cases.

**Figure 2:**
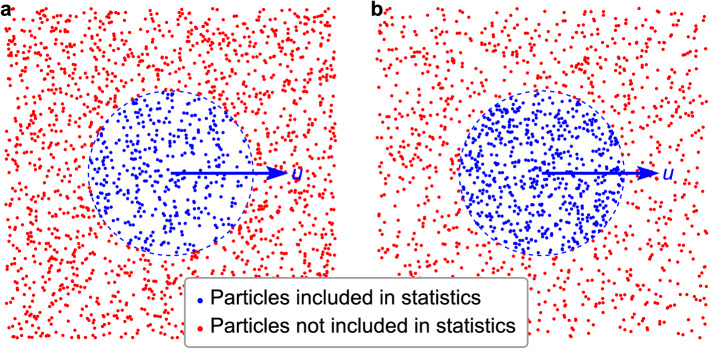
Relationships between the target (sub-) particles/domain and the total particles/domain. (**a**), The constrained state of $\text {I}u$: the number of blue particles follows the Poisson distribution based on time with the same strength as the Poisson distribution of the red particles based on location. (**b**), The constrained state of ${\text {III}}u$: the number of blue particles is fixed.

These special stochastic-constrained particle swarms, as a sub-particle swarm of the total particle swarm in a completely random state, may be in a variety of different constrained states observed from the total particle swarm. In a certain period and a fixed target domain (the volume is fixed and the location can move with the average velocity of the target particle swarm, the same is done below), when a sub-particle swarm is in a completely random (free) state, the location distribution of the particles in that state follows the Poisson distribution based on time with the same strength as the Poisson distribution of the population based on location. The velocity direction distribution is also consistent with the population (the norm of the average velocity follows the same Maxwell distribution). When a sub-particle swarm remains in a special accidental state for a certain period, it is equivalent to the sub-particle swarm being subject to some constraints and being in a non-completely random state. According to the constraint situation of the sub-particle swarm, we categorize it into the following three types of constrained states: For the first type of constrained state, in a certain period and a fixed target domain, the location distribution of the particles follows a Poisson distribution based on time with the same strength as the Poisson distribution of the population based on location, but the norms of the average velocities do not follow the Maxwell distribution. The special case of this state is that the average velocity norms of all counted particles are constant at *u* under the condition that the location distribution remains unchanged, which is termed $\text {I}u$ (Fig. 2a). For the second type of constrained state, in a certain period and a fixed target domain, the norms of the average particle velocities follow the Maxwell distribution, but the location distribution of the particles in the domain does not follow the Poisson distribution based on time with the same strength as the Poisson distribution of the population based on location. The special case of this state is that the number of particles in the fixed target domain is fixed under the condition that the velocity direction distribution remains unchanged. For the third type of constrained state, in a certain period and a fixed target domain, the norms of the average particle velocities do not follow the Maxwell distribution, and the location distribution of the particles in the domain does not follow the Poisson distribution based on time with the same strength as the Poisson distribution of the population based on location. The special case of this state is that the number of particles is fixed and the average velocity norm of all particles is fixed as *u* in the fixed target domain, which is termed ${\text {III}}u$ (Fig. 2b). The abovementioned sub-particle swarm (${\mathcal {R}}_u$) with a constant average velocity during a certain period belongs to ${\text {III}}u$.

When a sub-particle swarm in the constrained state of ${\text {III}}u$ (${\mathcal {R}}_u$ or the target domain) is observed in the total domain (${\mathcal {R}}_0$), it has the characteristics of location aggregation and velocity direction aggregation, which influence the diffusion rate constant of the particles. Therefore, the dynamic phenomena of this type of particle swarm exhibits certain special properties. Essentially, these stochastic-constrained particles are biased random particles. Regarding biased random particles, previous studies have mainly focused on physics, chemistry and biology fields in various forms^7^. And the study for the motion of these biased random particles in astrophysics are usually regarding them as the multi-body interaction involving gravitation^8^. However, the motion form without considering gravitation has not been provided. In the past, the formula for the total mass distribution of galaxies or galaxy clusters, such as the Navarro–Frenk–White (NFW) profile^9^ and Einasto profile^10^, was derived based on experience. However, a formula regarding the most basic dynamics of randomly moving particles is lacking.

This study focused on the particle swarm in the constrained state of ${\text {III}}u$, deduced the diffusion equation of the particles in this case and identified the formation conditions of a non-diffusion particle swarm. The basic structure of this study is as follows. The mathematical model was deduced step-by-step based on the defined physical model. Before derivation, two verifications were performed. First, it was confirmed that the physical model contained special relativistic effects; second, the form of Schrödinger equation was derived from the physical model under certain conditions. The process of the two checks also clarified how to derive the mathematical model, that is, the generalized diffusion equation. The process of deriving the generalized diffusion equation includes the following: ($\textbf{i}$) vector decomposition. The decomposition of nonmoving particles in space is extended to the decomposition of a 2-dimensional vector representing the sum of the 3-dimensional vector of moving particles at a certain point in space, which forms the core of the whole derivation. ($\textbf{ii}$) The classic diffusion coefficient is reinterpreted and the essential key information is obtained. ($\textbf{iii}$) Based on ($\textbf{i}$) and ($\textbf{ii}$), the equations are conjoined according to the classical diffusion principle to obtain the generalized diffusion equation. Furthermore, certain important parts related to the equation are discussed and verified. The following is a detailed description.

## Methods

In this study, a mathematical model was obtained through the logical derivation based on a physical model. Mathematica 13.1.0 for Mac (*Wolfram Research Inc*.) was used for all of the mathematical calculations, and the hardware was a Mac mini (Z12P) with the macOS Monterey 12.3.1 operating system. The solutions to each of the specific problems can be found in the Supplementary Information.

## Results and discussions

### Physical model

It is assumed that there are countless identical point particles with certain masses in an infinite 3-dimensional space. Their speed is *c*, the motion directions of each particle are evenly distributed in a 3-dimensional space, and there is no interaction between these particles. Our research object is a subset of such particles. The particles in this subset are in the special case of the third type of constrained state (i.e., ${\text {III}}u$, the blue domain in Fig. 2b) observed from the total domain.

### Special relativistic effects in the constrained state of I*u*

In this study, the “point particles” described above are called “particles” or “1-particles”, whereas the larger finite-mass-level particles composed of *k* particles are called “*k*-particles”. The *k*-particles or aggregates mentioned in this section are *k*-generalized-particles or aggregates. And the *k*-particle term implies that only *k* particles are counted, irrespective of whether they are truly clustered or not. The 1-particles can be represented by random vectors with equal norms that are equal to the same movement speeds in Euclidean space. Thus, the “random vectors” and “randomly moving particles (or velocities)” mentioned in this study have the same meaning.

My previous study^6^ has proven that the vector group in the constrained state of $\text {I}u$ formed by random vectors with equivalent norms has a special relativistic effect. That is, because of the statistical effect, when the centroid of the sub-particle swarm moves at a speed of *u* in one direction, the particles or the generalized *k*-particles formed by the sub-particles will lose a certain degree of freedom in other directions (or in other words, the movement trends in other directions decrease), resulting in the effect of special relativity. Here, the slowing ratio $\dfrac{\sqrt{c^2-u^2}}{c}$ of the particles in ${\mathcal {R}}_u$ or generalized aggregates they form is recorded as $\Gamma$[$\cdot$] or $\Gamma$ (we call it the $\Gamma$, or $\Gamma$[$\cdot$], effect). Although the particles in ${\mathcal {R}}_u$ are in the constrained state of $\text {I}u$ when observed from ${\mathcal {R}}_0$, they are in a completely random state when observed from ${\mathcal {R}}_u$. Moreover, my previous study^6^ has confirmed that all the physical laws are identical to that of the case while studying a *k*-generalized-particle in ${\mathcal {R}}_0$ observed from ${\mathcal {R}}_0$ and in ${\mathcal {R}}_u$ observed from ${\mathcal {R}}_u$. In the constrained state of $\text {I}u$, the particles themselves or the generalized particles formed by the particles show the effect of special relativity due to the aggregation effect of velocity direction; in the constrained state of ${\text {III}}u$, the aggregation effect also includes location aggregation (however, the two aggregation effect are uncorrelated to each other). Here, these two (aggregation) effects combined with the simultaneous effects of the velocity direction and location aggregation (such particles are in the constrained state of ${\text {III}}u$) are collectively called the statistical effect of randomly moving particles. When these statistical effects work in tandem, the generation conditions of a non-diffusion particle swarm can be obtained. This is explained in detail below.

### Establishment of the vector diffusion equation in the constrained state of I*u*

According to the discussions in section "Special relativistic effects in the constrained state of I*u*", observing these stochastic-constrained particles from the constrained particle swarms cannot correctly perceive these constrained phenomena. Therefore, the constrained states mentioned below all imply observing from their background domains (${\mathcal {R}}_0$). Irrespective of the movements of these particles in 3-dimensional space, their trajectories are continuous, which leads to diffusion (or agglomeration) behavior, which is the generalized diffusion of randomly moving particles in the constrained state of ${\text {III}}u$. Considering particles of the same mass and speed, the generalized diffusivity of the corresponding random vectors is equivalent to the generalized diffusivity of random momenta (which are also vectors). It is considered that the scale of the “generalized diffusivity of vectors” is simply the scale that is most suitable for describing the invariant laws for randomly moving particles. More information will be lost if the scale is even slightly more macroscopic (e.g., the scale can be approximately described by real diffusion), and there will be no invariant statistical law to follow if the scale is even slightly more microscopic (e.g., the scale described at the beginning of this paragraph). At this scale, the external behavior of the vectors in a tiny space cannot be considered isotropic. Before studying the particles in the constrained state of ${\text {III}}u$, we first study the particles in the constrained state of $\text {I}u$. Temporarily, the $\Gamma$ effect is not considered here; thus, it is consistent with the scenario of a completely free state. Compared with the ${\text {III}}u$ case, there is only diffusion without agglomeration in $\text {I}u$, and the other cases are consistent. In the constrained state of $\text {I}u$ (not considering the $\Gamma$ effect) observed from ${\mathcal {R}}_0$, the total vector in a certain domain always points in an uncertain direction, and the norm is directly proportional to *k*, where *k* is the number of vectors (see Part 1 of the Supplementary Information for details). Although the direction of the total vector in a tiny space cannot be determined, we hope to use appropriate constraints to obtain the distribution rules governing the norm and direction of the total vector at any location in space.

First, we determine the constraints acting on spatial vectors (norms and directions). Let the density of the vector sum at some point ${\mathcal {P}}$ in space be denoted by $\varvec{{\mathcal {X}}}$, which is a function of location and time, that is, $\varvec{{\mathcal {X}}}(x, y, z, t)$. It is defined as follows: at a certain time *t*, let $\varvec{{\mathcal {Y}}}({\mathcal {V}})$ be a function of the sum of all vectors in the closed domain ${\mathcal {V}}$ containing ${\mathcal {P}}(x, y, z)$; and $\varvec{{\mathcal {X}}}(x, y, z, t)=\underset{{{\mathcal {V}} \rightarrow {\mathcal {P}}}}{\lim }\dfrac{\varvec{{\mathcal {Y}}}({\mathcal {V}})}{{\mathcal {V}}}$ [in the following, $\varvec{{\mathcal {X}}}$ is also a function of the spatial coordinates (*x*, *y*, *z*) and the time coordinate *t*].

**Figure 3:**
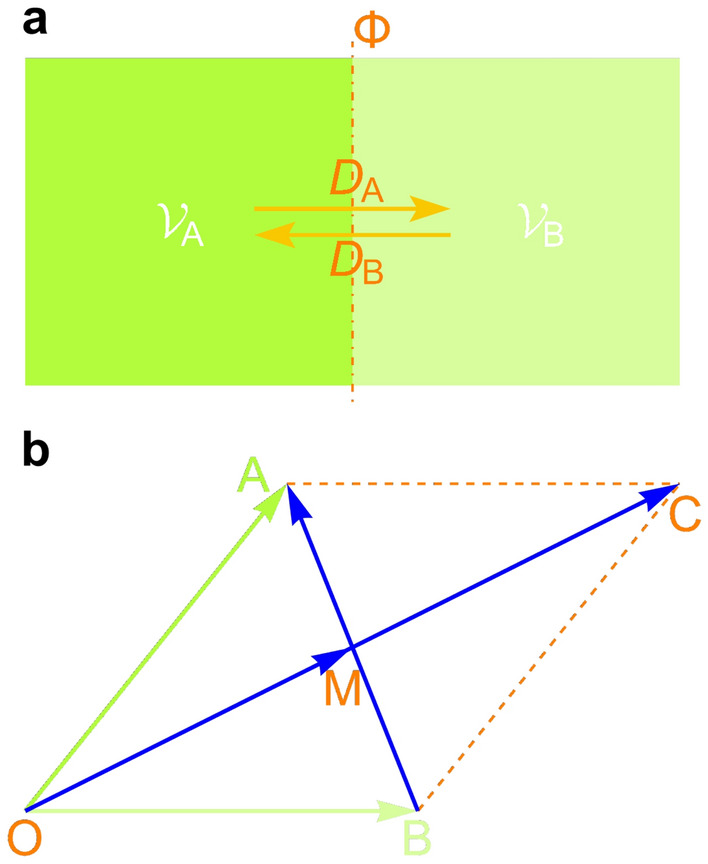
Illustration of the principle of the generation of a mutual diffusion potential in microdomains ${\mathcal {V}}_{\textrm{A}}$ and ${\mathcal {V}}_{\textrm{B}}$. (${{\textbf {a}}}$), Illustration of diffusion potential. (${{\textbf {b}}}$), Vector representation of diffusion potential.

$\varvec{{\mathcal {X}}}$ is the statistical average vector. The relationship between $\varvec{{\mathcal {X}}}$ and the number of vectors follows a distribution. As illustrated in Fig. 3a, it is assumed that there are two microdomains ${\mathcal {V}}_{\textrm{A}}$ and ${\mathcal {V}}_{\textrm{B}}$ of the same size along the normal direction on both sides of the segmentation surface $\Phi$. If the sum of all vectors in ${\mathcal {V}}_{\textrm{A}}$ is $\mathop {\mathrm{\mathrm OA}}\limits ^{\rightharpoonup }$ and the sum of all vectors in ${\mathcal {V}}_{\textrm{B}}$ is $\mathop {\mathrm{\mathrm OB}}\limits ^{\rightharpoonup }$, then their sum is $\mathop {\mathrm{\mathrm OC}}\limits ^{\rightharpoonup }$, and their difference is $\mathop {\mathrm{\mathrm BA}}\limits ^{\rightharpoonup }$. Let the sum and difference vectors intersect at point M (Fig. 3b). Because the velocity direction distribution is homogeneous and there is no need to consider the statistical effects due to location aggregation here, considering the previous assumption that the domains ${\mathcal {V}}_{\textrm{A}}$ and ${\mathcal {V}}_{\textrm{B}}$ on both sides of $\Phi$ are equal, after the particles randomly move and mix, both vectors must tend to approach their average value $\mathop {\mathrm{\mathrm OM}}\limits ^{\rightharpoonup }$; that is, both $\mathop {\mathrm{\mathrm OA}}\limits ^{\rightharpoonup }$ and $\mathop {\mathrm{\mathrm OB}}\limits ^{\rightharpoonup }$ tend toward $\mathop {\mathrm{\mathrm OM}}\limits ^{\rightharpoonup }$ (this is a diffusion potential across a membrane. Scalar concentration difference can generate concentration gradient, and vector difference can generate vector gradient. Their essence is the random motion of particles). The change rate of $\mathop {\mathrm{\mathrm OA}}\limits ^{\rightharpoonup }$ or $\mathop {\mathrm{\mathrm OB}}\limits ^{\rightharpoonup }$ to $\mathop {\mathrm{\mathrm OM}}\limits ^{\rightharpoonup }$ depends on the difference between $\mathop {\mathrm{\mathrm OA}}\limits ^{\rightharpoonup }$ and $\mathop {\mathrm{\mathrm OB}}\limits ^{\rightharpoonup }$ and the diffusion (motion) rate of particles. Accordingly, the rate of change in $\varvec{{\mathcal {X}}}$ along the normal direction (the motion or the vector generated by the motion in the other two tangent directions is invalid) at a particular point should be related to the time-dependent rate of change in $\varvec{{\mathcal {X}}}$ in defined domain. This time-dependent rate of change is also influenced by another inherent factor (i.e., the velocity of the particles forming $\varvec{{\mathcal {X}}}$), the concrete value of which is temporally uncertain. Therefore, the above two rates of change should be directly proportional when the differences between particles caused by density (location aggregation of particles) are neglected.

**Figure 4:**
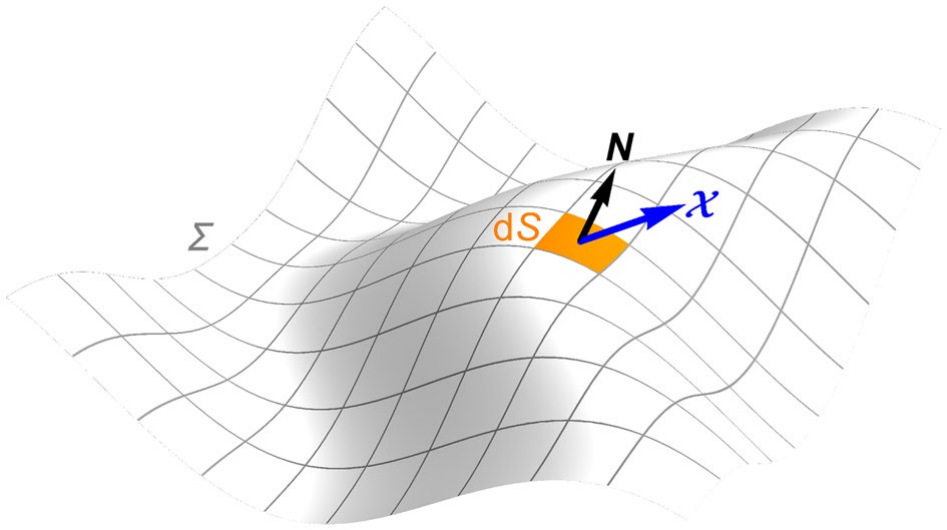
Illustration of the diffusion of the vector sum density $\varvec{{\mathcal {X}}}$.

If a domain ${\mathcal {W}}$ is enclosed by a closed surface $\it \Sigma$, then during the infinitesimal period $\textrm{d}t$, the directional derivative $\dfrac{\partial \varvec{{\mathcal {X}}}}{\partial {\varvec{N}}}$ of $\varvec{{\mathcal {X}}}$ along the normal direction of an infinitesimal area element $\textrm{d}S$ on the surface $\it \Sigma$ is directly proportional to the vector $\textrm{d}\varvec{{\mathcal {X}}}$ flowing through $\textrm{d}S$ along the normal direction in the closed domain ${\mathcal {W}}$ enclosed by $\it \Sigma$ (Fig. 4), under the assumption that the coefficient is a positive real number *D*.

For the time interval, $t_{\textrm{a}}$ to $t_{\textrm{b}}$, when the influence of the vector density on *D* is not considered (i.e., the diffusion coefficient is the same at every location), the variation of the vector sum $\varvec{{\mathcal {A}}}$ inside the closed surface $\it \Sigma$ is given as1According to the Gaussian formula, Eq. (1) can also be written in the form

2 $$\begin{aligned} \delta \varvec{{\mathcal {A}}}=\int _{t_{\textrm{a}}}^{t_{\textrm{b}}} \left( \iiint \limits _{{\mathcal {W}}}D\nabla ^2\varvec{{\mathcal {X}}}\textrm{d}x\textrm{d}y\textrm{d}z\right) \textrm{d}t, \end{aligned}$$

where $\nabla$ is the Hamilton operator, which describes the first derivative with respect to location (*x*, *y*, *z*). The left-hand side of Eq. (1) ($\delta \varvec{{\mathcal {A}}}$) can also be written as

3 $$\begin{aligned} \delta \varvec{{\mathcal {A}}}=\iiint \limits _{{\mathcal {W}}}\left( \int _{t_{\textrm{a}}}^{t_{\textrm{b}}}\frac{\partial \varvec{{\mathcal {X}}}}{\partial t}\textrm{d}t\right) \textrm{d}x\textrm{d}y\textrm{d}z. \end{aligned}$$

By setting the right of Eq. (3) equal to the right of Eq. (2) and transforming the order of integration, we can obtain

4 $$\begin{aligned} \int _{t_{\textrm{a}}}^{t_{\textrm{b}}}\!\iiint \limits _{{\mathcal {W}}}\frac{\partial \varvec{{\mathcal {X}}}}{\partial t}\textrm{d}x\textrm{d}y\textrm{d}z\textrm{d}t=\int _{t_{\textrm{a}}}^{t_{\textrm{b}}}\!\iiint \limits _{{\mathcal {W}}}D\nabla ^2\varvec{{\mathcal {X}}}\textrm{d}x\textrm{d}y\textrm{d}z\textrm{d}t. \end{aligned}$$

Based on the observation that $t_{\textrm{a}}$, $t_{\textrm{b}}$ and domain ${\mathcal {W}}$ are arbitrary, the following equation can be written as

5 $$\begin{aligned} \frac{\partial \varvec{{\mathcal {X}}}}{\partial t}=D\nabla ^2\varvec{{\mathcal {X}}}. \end{aligned}$$

To facilitate the task of vector decomposition in the constrained state of ${\text {III}}u$, a 3-dimensional vector needs to be converted into a plane vector. Next, we determine the constraints acting on plane vectors. Although the operation in Eq. (5) is performed using 3-dimensional vectors, when differential operations are performed on a spatial vector, the (sum or) difference operations are always performed at two points on the vectors that are separated by an infinitesimal distance; thus, all 3-dimensional vectors can exhibit only relative 2-dimensional characteristics. Consequently, by solving this differential equation, only 2-dimensional constraints can be obtained. Therefore, only the derivatives of plane vectors are needed to act as the derivatives of the 3-dimensional vectors (in this case, plane vectors can retain the important information, such as the norms of the vectors and the included angle between them). Moreover, according to the Sturm–Liouville theory, the function of plane vectors obtained by solving the partial differential equation expressed in terms of plane vectors is unique and corresponds to the 3-dimensional vectors obtained from a differential equation of the same form. It is assumed that the function of plane vectors describing the density of the vectors or momenta is $\varvec{{\mathcal {M}}}(x, y, z, t)$, which corresponds to $\varvec{{\mathcal {X}}}$ at the point (*x*, *y*, *z*, *t*) [unless otherwise stated, in the following, $\varvec{{\mathcal {M}}}$ is a function of the spatial coordinates (*x*, *y*, *z*) and the time coordinate *t*]. Thus, $\varvec{{\mathcal {X}}}$ can be replaced with $\varvec{{\mathcal {M}}}$. Following this replacement, it is obvious that the norm of the plane vector remains constant while its direction is reoriented. Finally, Eq. (5) can be written as

6 $$\begin{aligned} \left\| \frac{\partial \varvec{{\mathcal {M}}}}{\partial t}\right\| =D\left\| \nabla ^2\varvec{{\mathcal {M}}}\right\| . \end{aligned}$$

Now, let us determine the constraints on the direction of the plane vector $\varvec{{\mathcal {M}}}$. In view of the continuity of the trajectories of point particles, since $\varvec{{\mathcal {M}}}$ is also characterized in terms of the statistical properties of an enormous number of particles, it should also be smooth. According to the theory of plane curves, the first and second derivatives of a plane vector in any direction in space are vertical. If an equation relating these derivatives is established according to the above derivative relationship (Eq. 6), the direction needs to be adjusted to be consistent; otherwise, the equations cannot be equal; then, the unique and definite relationship can be written in the form

7 $$\begin{aligned} \frac{\partial \varvec{{\mathcal {M}}}}{\partial t}={\textbf{i}}\,D\nabla ^2\varvec{{\mathcal {M}}}, \end{aligned}$$

where ${\textbf{i}}$ is an imaginary unit. By multiplying both sides of Eq. (7) by ${\textbf{i}}$, the form of the Schrödinger equation (without an external field) can be obtained as

8 $$\begin{aligned} {\textbf{i}}\,\frac{\partial \varvec{{\mathcal {M}}}}{\partial t}=-D\nabla ^2\varvec{{\mathcal {M}}}. \end{aligned}$$

Equation (8) describes the distribution of a moving particle swarm (including the direction of movement) in the constrained state of $\text {I}u$ (not considering the $\Gamma$ effect) or in a completely free state following the same diffusion coefficient; in other words, it is the stochastic-constrained vector diffusion equation observed from ${\mathcal {R}}_0$. When *u* is small, the constrained state of $\text {I}u$ can also be approximated to a completely free state (the $\Gamma$ effect can be ignored) and Eq. (8) levels off to the Schrödinger equation without an external field. However, when *u* is large and there is both a location-constrained state (i.e., the constrained state of ${\text {III}}u$), the effect on diffusion is not clear. To more comprehensively describe this type of diffusion process (which is called generalized diffusion), further analysis is needed.

### Construction of the generalized diffusion equation in the constrained state of ${\text {III}}u$

To construct the generalized diffusion equation in the constrained state of ${\text {III}}u$, we need to consider several aspects, including whether the generalized diffusion coefficient *Ɖ* should vary and how to describe it to include the characteristics of the two types of constrained states.

When particles are in the constrained state of $\text {I}u$ (not considering the $\Gamma$ effect), they follow a diffusion equation with the same diffusion coefficient. However, when such particles are in the constrained state of ${\text {III}}u$, the effect of location aggregation on *Ɖ* should be considered, and *Ɖ* should vary with the value of the target vector. Suppose that, as illustrated in Fig. 3a, the vector sum density in the microdomain ${\mathcal {V}}_{\textrm{A}}$ is greater than that in the ${\mathcal {V}}_{\textrm{B}}$. If both cases (${\mathcal {V}}_{\textrm{A}}$ and ${\mathcal {V}}_{\textrm{B}}$) are in the constrained state of ${\text {III}}u$, there is a greater consumption of degrees of freedom for the higher density in the ${\mathcal {V}}_{\textrm{A}}$. In terms of probability, less uncertainty is introduced into the unit volume, which inevitably affects the (average) particle speed. Therefore, the overall particle speed in the ${\mathcal {V}}_{\textrm{A}}$ decreased. As mentioned above (or in Eq. 27 below), the particle speed is what determines *D*; therefore, the law governing the diffusion rate towards the right ($D _{\textrm{A}}$) is not identical to the law governing the diffusion rate in the ${\mathcal {V}}_{\textrm{B}}$ towards the left ($D _{\textrm{B}}$) (under the assumption that *Ɖ* is a combination of $D _{\textrm{A}}$ and $D _{\textrm{B}}$). Therefore, it is necessary for the generalized diffusion coefficient to vary in time with the vector sum density to reflect this inequality.

In view of the above considerations, choosing the appropriate quantitative function to describe this phenomenon (with different laws) is the key issue to be addressed in this study. First, the sum of the momentum vectors in the microdomain is decomposed, as described in the following subsection.

#### Vector decomposition

First, let us determine the distribution function for a certain number of nonmoving particles with equal probability (randomly) distributed in a certain domain, as follows: Suppose that the entire domain contains *n* particles in total. For convenience of description, the entire domain is also partitioned into *n* boxes of equal size. The gaps between the boxes and the wall thickness are both 0. This is a localized system. Now, let us determine the probability of *k* ($k\in {\mathbb {N}}_+$; the same is given below) particles in a local area containing ${\mathcal {M}}$ boxes (suppose that the particles are small enough to fall into the box, not the wall). In view of the statement described above, the probability of particles existing in each domain is the same. Accordingly, the total number of possible cases describing how $n$ particles can be randomly distributed among *n* boxes is $n^n$, there are $\genfrac(){0.0pt}0{n}{k}$ total ways that *k* particles can be randomly chosen from among *n* particles, there are ${\mathcal {M}}^k$ total ways in which the $k$ chosen particles can be randomly distributed among ${\mathcal {M}}$ boxes, and there are $\left( n-{\mathcal {M}}\right) ^{n-k}$ total ways in which the remaining $n-k$ particles can be randomly distributed among the remaining $n-{\mathcal {M}}$ boxes. Therefore, the probability $P({\mathcal {M}}, k)$ of *k* particles existing in ${\mathcal {M}}$ boxes can be expressed as

9 $$\begin{aligned} P({\mathcal {M}}, k)=\frac{\genfrac(){0.0pt}0{n}{k}{\mathcal {M}}^k\left( n-{\mathcal {M}}\right) ^{n-k}}{n^n}. \end{aligned}$$

Suppose that the number *n* of particles in the entire domain is infinite; then, by taking the limit of Eq. (9) as $x \rightarrow +\infty$, we find that

10 $$\begin{aligned} P({\mathcal {M}}, k)=\frac{\mathrm e^{-{\mathcal {M}}}{\mathcal {M}}^k}{k!}, \end{aligned}$$

again, where ${\mathcal {M}}$ denotes the number of boxes comprising the local domain of interest (the size of the volume in 3-dimensional space), *k* denotes the number of particles in that domain of ${\mathcal {M}}$ boxes, and *P* denotes the probability that *k* particles exist in that domain. Equation (10) is the (location-based) Poisson distribution.

It is considered that this is the most appropriate method of partitioning a whole domain (the domain can be the whole universe or merely a broad range including the objects of investigation) into uniform boxes with the same number as that of particles. Besides reducing the parameters involved and facilitating discussion, the reasons are as follows: if the boxes are slightly larger, they will not ensure the accuracy of the following vector decomposition; if they are slightly smaller, they will not adequately reflect the grouping effect of the particles. Therefore, in this study, the whole domain is divided into a number of uniform boxes equal to the number of particles it contains, and this partitioning serves as the basis for all of the following discussions. In this study, the whole domain (environment) is called the T-domain (it is the sub-domain of sub-domain in Fig. 1), and the local domain (target) is called the S-domain; the set of all particles contained in the T-domain is called the T-particle swarm (it is the sub-particle swarm of sub-particles as shown in Fig. 2), and the subset of particles contained in the S-domain is called the S-particle swarm.

Next, we will investigate the equiprobability distribution of the nonmoving particle swarm in the abovementioned S-domain ${\mathcal {V}}$. In Eq. (10), ${\mathcal {M}}$ denotes the number of boxes (volume) spanned by certain S-domain (which belonged to the domain in which the target particles are distributed). Put another way, when the T-domain is partitioned into uniform boxes following the above method, ${\mathcal {M}}$ can also denote the average relative density of the particles in the S-domain ${\mathcal {V}}$, where the reference density is the average density of the T-particle swarm in the T-domain. ${\mathcal {M}}$ represents the corresponding multiple of the average density, *k* denotes the number of particles in one box, and *P* is the probability of *k* particles existing in that box. Thus, the distribution of the S-particle swarm in ${\mathcal {V}}$ is a Poisson distribution with density intensity ${\mathcal {M}}$. Next, we will analyze the Poisson distribution formula given in Eq. (10). In fact, it is the proportion of each term determined by *k* (when $\mathrm e^{{\mathcal {M}}}$ is expanded as a power series) to the value of $\mathrm e^{{\mathcal {M}}}$. The meaning here is that it is also the proportion of the number of boxes containing *k* particles each to the total number of boxes in ${\mathcal {V}}$ when the S-particle swarm of relative density ${\mathcal {M}}$ is distributed among the reference boxes determined by the above criteria and spanned by the S-domain ${\mathcal {V}}$ (assuming that the number of boxes spanned by ${\mathcal {V}}$ is sufficiently large). According to mathematical analysis, we can see that the power series expansion for this case is unique, and obviously, this ratio distribution is also unique. If the right-hand side of Eq. (10) is multiplied by *k*, the result, denoted by $R({\mathcal {M}}, k$), takes the following form:

11 $$\begin{aligned} R({\mathcal {M}}, k)=\frac{\mathrm e^{-{\mathcal {M}}}{\mathcal {M}}^k}{(k-1)!}. \end{aligned}$$

In this way, termwise addition (by *k*) based on this expression offers a possible form for the decomposition of ${\mathcal {M}}$ into infinite items. Because the power series expansion above is unique, this decomposition form of the containing power series is also unique. According to the previous statement of physical meaning, the meaning of Eq. (11) is the relative density contributed by the particles in the boxes that contain *k* particles each to the total relative density ${\mathcal {M}}$ (the average relative density in ${\mathcal {V}}$) after the particles of relative density ${\mathcal {M}}$ are dispersed among the (infinitely many) reference boxes spanned by ${\mathcal {V}}$ with equal probability. Multiplying Eq. (11) by the number of boxes contained in ${\mathcal {V}}$ yields the total number of particles in the boxes containing *k* particles each. Since the distribution of particles in this form is definite (following the Poisson distribution), from this point of view, the decomposition of the relative density ${\mathcal {M}}$ in this (containing power series) form is also unique.

If $\varvec{{\mathcal {M}}}$ is a complex number (or plane vector), Eq. (11) can be written in vector form as follows:

12 $$\begin{aligned} R(\varvec{{\mathcal {M}}}, k)=\frac{\mathrm e^{-\varvec{{\mathcal {M}}}}\varvec{{\mathcal {M}}}^k}{(k-1)!}. \end{aligned}$$

The form obtained by dividing Eq. (12) by *k* yields still the ratio of each term (complex) determined by *k* (when $\textrm{e}^{\varvec{{\mathcal {M}}}}$ is expanded as a power series) to the complex of $\textrm{e}^{\varvec{{\mathcal {M}}}}$. There is one more dimension here, and the power series expansion is still unique. Similarly, the termwise addition of Eq. (12) also provides a decomposition form for the vector $\varvec{{\mathcal {M}}}$. This decomposition form of the containing power series is also unique.

**Figure 5:**
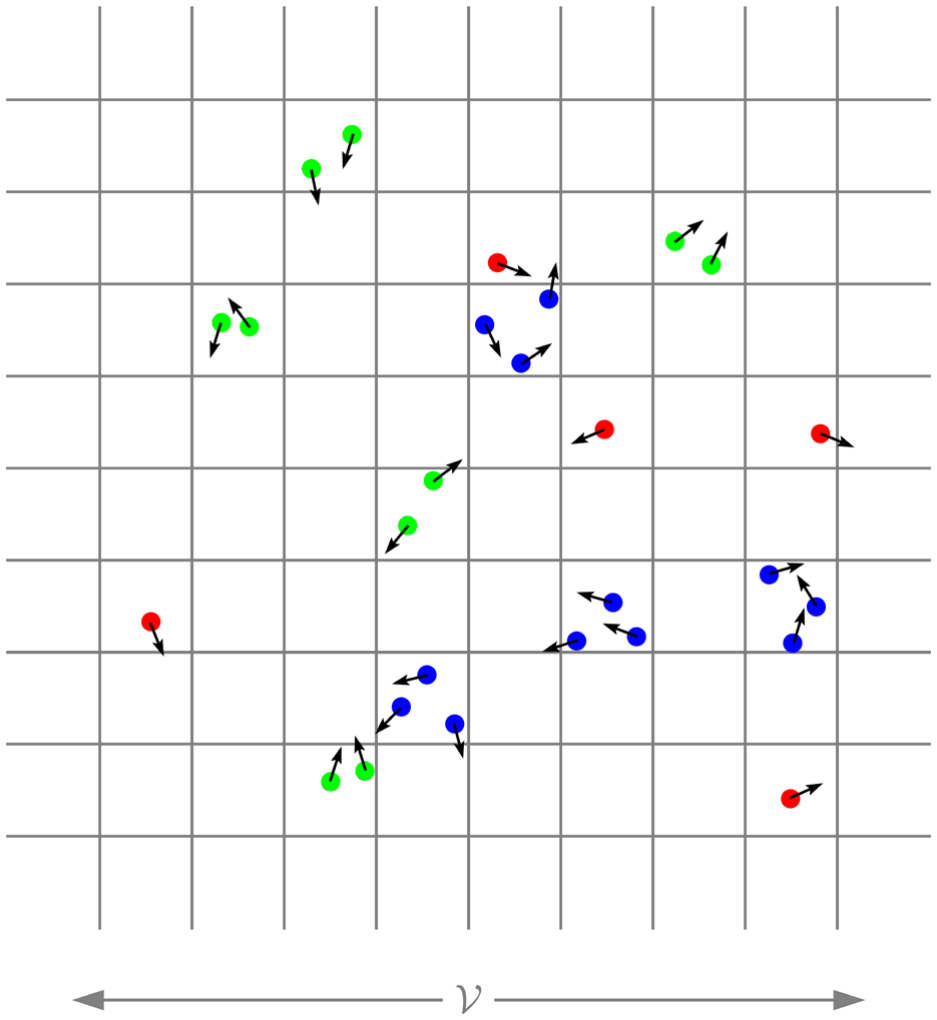
Illustration of the physical meaning of $\varvec{{\mathcal {Y}}}_k$ ($k=1, 2, 3, \ldots$) in the S-domain ${\mathcal {V}}$ (a planar figure is used to represent the stereo figure). The vector sum of the red particles ($k=1$) is $\varvec{{\mathcal {Y}}}_1$, the vector sum of the green particles ($k=2$) is $\varvec{{\mathcal {Y}}}_2$, and the vector sum of the blue particles ($k=3$) is $\varvec{{\mathcal {Y}}}_3$, $\cdots$.

Now, we study the distribution of the velocity of the moving S-particle swarm in the abovementioned S-domain ${\mathcal {V}}$. If the particles in the T-particle swarm move randomly in the T-domain, the distribution of the S-particle swarm in one time slice in a sufficiently small S-domain (when the particle speed is fast enough) can also be approximated as an equiprobable distribution. At the human scale—and it will be proven with self-consistency that, in fact, the same obtains at any scale—the number of S-particles in almost every “microdomain” of the universe can be regarded as approaching infinity; therefore, the number distribution of particles in the moving S-particle swarm in a certain microdomain ${\mathcal {V}}$ can be described by Eq. (10). The moving particles in each type of box partitioned by $k$ in one S-domain ${\mathcal {V}}$ can form a component vector (denoted by $\varvec{{\mathcal {Y}}}_k$, as shown schematically in Fig. 5), and these components can be added together to generate the total 3-dimensional vector $\varvec{{\mathcal {Y}}}$ in ${\mathcal {V}}$, that is

13 $$\begin{aligned} \varvec{{\mathcal {Y}}}=\sum _{k=1}^{\infty }\varvec{{\mathcal {Y}}}_k. \end{aligned}$$

Once $\varvec{{\mathcal {Y}}}$ formed by the moving S-particle swarm in ${\mathcal {V}}$, which includes the specific number of (equivalent) particles, is determined (i.e., the average speed *u* of the S-particles or T-particles is determined observed from ${\mathcal {R}}_0$), the norm (mathematical expectation) of each component vector should be (approximately) directly proportional to the number of particles forming it when the number of particles is large (see Part 1 of the Supplementary Information for details). Note that the number of samples in ${\mathcal {V}}$ is very large even when $k=1$. Therefore, the ratios between the norms (mathematical expectations) of the component vectors in various boxes partitioned by *k* are uniquely determined by the form of (containing) the power series determined by Eq. (11) (observed from ${\mathcal {R}}_0$). In other words, when ${\mathcal {M}}$ represents the relative density of the particles in ${\mathcal {V}}$, we have the following relationship:

14 $$\begin{aligned} \left\| \varvec{{\mathcal {Y}}}_1\right\| :\left\| \varvec{{\mathcal {Y}}}_2\right\| :\cdots =R({\mathcal {M}}, 1):R({\mathcal {M}}, 2):\cdots . \end{aligned}$$

As the limiting value $\varvec{{\mathcal {X}}}$ of the quotient of $\varvec{{\mathcal {Y}}}$ and ${\mathcal {V}}$, it can still be considered as a sum of 3-dimensional vectors in the S-domain ${\mathcal {V}}$. Therefore, there is also a form of component vectors with the ratios of norms determined by Eq. (11) spanning various boxes partitioned by *k*. When the 3-dimensional component vectors (spanning various boxes partitioned by *k*) of the 3-dimensional vector $\varvec{{\mathcal {X}}}$ are mapped to the 2-dimensional component vectors (spanning various boxes partitioned by *k*) of the plane vector $\varvec{{\mathcal {M}}}$, it is clear that there is also a corresponding 2-dimensional form of component vectors with the ratios of norms determined by Eq. (11) (namely, the ratios of norms follow a Poisson distribution corresponding to the number of particles), but the direction is not determined. That is, when $\varvec{{\mathcal {X}}}_1$, $\varvec{{\mathcal {X}}}_2$, $\cdots$ represent the component vectors of $\varvec{{\mathcal {X}}}$ respectively and $\varvec{{\mathcal {M}}}_1$, $\varvec{{\mathcal {M}}}_2$, $\cdots$ represent the component vectors of $\varvec{{\mathcal {M}}}$ respectively, we have

15 $$\begin{aligned} \left\| \varvec{{\mathcal {Y}}}_1\right\| :\left\| \varvec{{\mathcal {Y}}}_2\right\| :\cdots =\left\| \varvec{{\mathcal {X}}}_1\right\| :\left\| \varvec{{\mathcal {X}}}_2\right\| :\cdots =\left\| \varvec{{\mathcal {M}}}_1\right\| :\left\| \varvec{{\mathcal {M}}}_2\right\| :\cdots . \end{aligned}$$

According to Eqs. (14) and (15), we can obtain the following relationship:

16 $$\begin{aligned} \left\| \varvec{{\mathcal {M}}}_1\right\| :\left\| \varvec{{\mathcal {M}}}_2\right\| :\cdots =R({\mathcal {M}}, 1):R({\mathcal {M}}, 2):\cdots . \end{aligned}$$

According to the conclusion in Part 1 of the Supplementary Information, the norm (mathematical expectation) of each component vector is the product of the number of particles forming it and the speed of the system it located observed from ${\mathcal {R}}_0$. Therefore, we obtain

17 $$\begin{aligned} \left\| \varvec{{\mathcal {M}}}\right\| ={\mathcal {M}}\cdot u. \end{aligned}$$

Note that when ${\mathcal {M}}$ represents a relative scalar, $\varvec{{\mathcal {M}}}$ represents a relative vector. Therefore, $\left\| \varvec{{\mathcal {M}}}\right\| ={\mathcal {M}}$ is always true when $u=1$, where *u* is the average speed of the T-particles. In this way,

18 $$\begin{aligned} \left\| \varvec{{\mathcal {M}}}_1\right\| :\left\| \varvec{{\mathcal {M}}}_2\right\| :\cdots =R(\left\| \varvec{{\mathcal {M}}}\right\| , 1):R(\left\| \varvec{{\mathcal {M}}}\right\| , 2):\cdots . \end{aligned}$$

In other words, when $u=1$, the ratios of norms of the component vectors of $\varvec{{\mathcal {M}}}$ are the ratios of the power series (determined by the Poisson distribution) forms of its own norm.

When $\varvec{{\mathcal {M}}}$ is decomposed into $\varvec{{\mathcal {M}}}_1$, $\varvec{{\mathcal {M}}}_2$, $\cdots$ denoted by itself (i.e., $u=1$), the relationship between $\left\| \varvec{{\mathcal {M}}}_1\right\|$, $\left\| \varvec{{\mathcal {M}}}_2\right\|$, $\cdots$ must satisfy Eq. (18). In view of the uniqueness of $R(\left\| \varvec{{\mathcal {M}}}\right\| , k)$, which is the power series form of the norms, $\varvec{{\mathcal {M}}}_k$ must be expressed in the form of $R(\varvec{{\mathcal {M}}}, k)$ (Eq. 12, or at least the form of $R(\varvec{{\mathcal {M}}}, k)\cdot \mathrm e^{\varvec{{\mathcal {M}}}}$) to satisfy Eq. (18). At this point, the direction of $\varvec{{\mathcal {M}}}_k$ is uniquely determined. In view of the termwise addition (by *k*) of Eq. (12) is the unique decomposition of $\varvec{{\mathcal {M}}}$; therefore, the plane mapping of the sum of all the vectors in the boxes containing the same number *k* of particles is the component vector determined by *k* in Eq. (12). When *k* takes all values in ${\mathbb {N}}_+$, the termwise sum of these terms is the unique decomposition of $\varvec{{\mathcal {M}}}$ (spanning various boxes partitioned by *k*), namely,

19 $$\begin{aligned} \varvec{{\mathcal {M}}}=\sum _{k=1}^{\infty }\frac{\mathrm e^{-\varvec{{\mathcal {M}}}}\varvec{{\mathcal {M}}}^k}{(k-1)!}. \end{aligned}$$

The above analysis shows that two conditions must be satisfied for $\varvec{{\mathcal {M}}}$ to be uniquely decomposed into components divided by *k*. On the one hand, $u=1$ (or $\left\| \varvec{{\mathcal {M}}}\right\| ={\mathcal {M}}$) must be satisfied; on the other hand, $\left\| \varvec{{\mathcal {M}}}\right\|$ must be a relative value as ${\mathcal {M}}$. Therefore, it is clear that $\varvec{{\mathcal {M}}}$ should also be a relative vector. Furthermore, $\varvec{{\mathcal {M}}}$ should be not only a multiple of the number of reference boxes but also a multiple of the speed of the system (that is, the norm of the average velocity of the counted particles; $u=1$ can be satisfied only if *u* is regarded as a relative value $u^*$). Therefore, the reference value of vector $\varvec{{\mathcal {M}}}$ is *nu* (where *u* is the absolute speed of the target domain in the background domain). Accordingly, $\varvec{{\mathcal {M}}}$ in section "Establishment of the vector diffusion equation in the constrained state of I*u*" should be exactly the relative vector sum density, which has the same direction as the absolute sum of the vectors located at that place observed from ${\mathcal {R}}_0$. As mentioned above, the sum and difference operations between two spatial vectors are performed in their shared plane. In this plane, they can be respectively decomposed into a sum of plane vectors, as described in Eq. (19). Therefore, the two sets of plane component vectors can also serve as their respective spatial component vectors to correspondingly perform sum, difference or derivative operations.

#### Description of diffusion

Suppose that the standard deviation of the projection (treated as a random variable; the same is done below) of the velocities of the *k* equivalent particles forming a *k*-particle (that is the *k*-generalized-particle; the same is done below) onto each equivalent coordinate axis is $\sigma$. As mentioned earlier, the speeds of *k*-particles follow the Maxwell distribution with scale parameter $\dfrac{\sigma }{\sqrt{k}}$ (When it is in the constrained state of $\text {I}u$ not considering the $\Gamma$ effect or in a completely free state, the speed of particle diffusion to uniform mixing in Fig. 3a is determined by the statistical average of the particle velocities, which is the inherent property of the system. Here, the particles in the target domain is regarded as a system with uniform distribution in the velocity direction, that is, the speeds of generalized particles follow the Maxwell distribution, and the average speed can be obtained according to the Maxwell distribution^6^). Then, the average speed of *k*-particles is

20 $$\begin{aligned} {\overline{v}}=2\sqrt{\frac{2}{\pi }} \cdot \frac{\sigma }{\sqrt{k}}. \end{aligned}$$

For $k_{\textrm{a}}$- and $k_{\textrm{b}}$-particles, the ratio of their average speeds is

21 $$\begin{aligned} \frac{\overline{v_{\textrm{a}}}}{\overline{v_{\textrm{b}}}}=\frac{\sqrt{k_{\textrm{b}}}}{\sqrt{k_{\textrm{a}}}}. \end{aligned}$$

Because the sizes, or masses, of all 1-particles (forming *k*-particles) are identical, if the masses of a $k_{\textrm{a}}$-particle and a $k_{\textrm{b}}$-particle are $m_{\textrm{a}}$ and $m_{\textrm{b}}$, respectively ($m\propto k$), then according to the relationship shown in Eq. (21), the ratio of their average speeds can also be written as

22 $$\begin{aligned} \frac{\overline{v_{\textrm{a}}}}{\overline{v_{\textrm{b}}}}=\frac{\sqrt{m_{\textrm{b}}}}{\sqrt{m_{\textrm{a}}}}. \end{aligned}$$

See Part 2 of the Supplementary Information for the detailed calculation and derivation process. According to Eq. (22), for any-particles, the product of the square root of mass and the average speed is a constant (suppose it is $\kappa _{\textrm{a}}$). Then, when the mass of a *k*-particle is *m*, its average speed is

23 $$\begin{aligned} {\overline{v}}=\frac{\kappa _{\textrm{a}}}{\sqrt{m}}. \end{aligned}$$

The diffusion coefficient can be defined as follows: it is the mass or mole number of a substance that diffuses vertically through a unit of area along the diffusion direction per unit time and per unit concentration gradient. Therefore, it is believed that classical real diffusion is consistent with the essence of vector diffusion described here (the two diffusions that are achieved both require the random displacement of *k*-particles). According to the Einstein–Brown displacement equation, the diffusion coefficient is

24 $$\begin{aligned} D=\frac{{\overline{x}}^2}{2t}, \end{aligned}$$

where ${{\overline{x}}}$ is the average displacement of *k*-particles along the direction of the *x*-axis. To replace the average displacement ${{\overline{x}}}$ in Eq. (24) with the average velocity (namely, $\overline{{\varvec{V}}}$) of *k*-particles along the 3-dimensional directions, this diffusion coefficient can be transformed into (in isotropic system)

25 $$\begin{aligned} D=\frac{\left\| \overline{{\varvec{V}}}\right\| ^2}{6}t^1, \end{aligned}$$

where $t^1$ and the *t* implied in $\left\| \overline{{\varvec{V}}}\right\| ^2$ are consistent, so $t^1=1$ s. The average speed $\overline{{\varvec{V}}}$ is related to the speed of a single *k*-particle. If the (average) speed of a single *k*-particle is ${\overline{v}}$, then the statistical average speed of these particles in one direction is

26 $$\begin{aligned} \left\| \overline{{\varvec{V}}}\right\| =\frac{{\overline{v}}}{2}. \end{aligned}$$

The *k*-particle swarm spreads in the plane at this rate. By substituting Eq. (26) into Eq. (25) and combining $t^1=1$ s into the coefficient, which we then denote by $\kappa _{\textrm{b}}$, we can obtain

27 $$\begin{aligned} D=\kappa _{\textrm{b}}\, {\overline{v}}^2, \end{aligned}$$

where $\kappa _{\textrm{b}}$ is a constant coefficient with units of seconds (s).

By substituting Eqs. (23) into (27), the diffusion coefficient of a (*k*-)particle swarm of (average) mass *m* is obtained:

28 $$\begin{aligned} D=\kappa _{\textrm{b}}\left( \frac{\kappa _{\textrm{a}}}{\sqrt{m}}\right) ^{\!2}=\frac{{\kappa _{\textrm{a}}}^{\!2}\kappa _{\textrm{b}}}{m}. \end{aligned}$$

In view of the diffusion coefficient *D* only affecting the diffusion rate, the above equation (Eq. 28) can also be thought of as the apparent diffusion coefficient of particle(s) with mass *m* described by the 1-particle swarm (which forms a particle of mass *m* after collapse) in the constrained state of $\text {I}u$. Here, we suppose that

29 $$\begin{aligned} {\kappa _{\textrm{a}}}^{\!2}\kappa _{\textrm{b}}=\frac{\hbar }{2}. \end{aligned}$$

As the situation in ${\mathcal {R}}_u$ observed from ${\mathcal {R}}_0$, *D* should also be affected by the $\Gamma$[$\cdot$] effect, which is abbreviated as

30 $$\begin{aligned} D=\frac{\hbar \, \Gamma ^2}{2\,m}. \end{aligned}$$

#### Construction of the generalized diffusion equation

Previously, we adopted the assumption that there is no interaction between point particles. Accordingly, in a time slice of a microdomain, the decomposition of the vector given by Eq. (19) must be exhibited observed from ${\mathcal {R}}_0$, and all boxes containing the same number of particles in different microdomains are equivalent. This is because there should be no differences between boxes of the same type (i.e., containing the same number of particles) when (the entire target domain is expressed as a system with a relative average speed of 1 and) the Poisson distribution determines the number differences of boxes of different types in different microdomains. Although the moving particles in the second or third constrained state can be distributed in a time slice of the microdomains with the same probability, when the overall behavior of *k* particles is counted, their average speed will inevitably slow down. Consequently, in a certain period, the location distribution of the particles does not follow the Poisson distribution based on time with the same strength as the poisson distribution for the population based on location, the “slow down” effect will be retained according to the location characteristics; in other words, the degrees of freedom of particles will be reduced or affected by the second or third type of constraint effect. The particles in various boxes partitioned by *k* move at their average relative speed, and the centroids of boxes containing *k* particles each are, on average, located at the center of each box. Among all boxes of the same type (i.e., containing *k* particles), the average relative speed of each *k*-particle is the same and must conform to the diffusion form of Eq. (8) determined by the diffusion coefficient for particles of this type. Therefore, according to the particle numbers *k* in the previously partitioned boxes, from 1 to $\infty$, we study the corresponding term $R(\varvec{{\mathcal {M}}}, k)$, which is the component vector of $\varvec{{\mathcal {M}}}$. First, we investigate the diffusion of individual terms, and then, we add them together to characterize the overall slowing behavior of diffusion.

Here, all the particles in each box containing *k* particles are regarded as forming a *k*-particle of a larger mass level, and together, all *k*-particles in all boxes containing *k* particles in microdomain ${\mathcal {V}}$ are called the *k*-particle swarm in that microdomain. Based on the above discussion, it can be considered that the average relative speed of each (*k*-)particle in the *k*-particle swarm is the same, and all of them have the same diffusion coefficient. According to the relationship given in Eq. (28) (the diffusion coefficient is inversely proportional to the mass of a *k*-particle, or the number of 1-particles forming a *k*-particle), if the diffusion coefficient of a 1-particle swarm is $D_1$, then the diffusion coefficient of a *k*-particle swarm is

31 $$\begin{aligned} D_k=D_1\cdot \frac{1}{k}, \end{aligned}$$

where $\dfrac{1}{k}$ is called the diffusion coefficient factor.

When the particles are in the constrained state of $\text {I}u$ or in a completely random state, the diffusion behavior of interest is that of a 1-particle swarm. It is consistent with the Schrödinger equation when the target particle swarm moves along the average speed of *u*. Therefore, the diffusion coefficient is

32 $$\begin{aligned} D_1=-\frac{\hbar \, \Gamma ^2}{2\,m}. \end{aligned}$$

The diffusion equation determined by this coefficient describes the dynamics of the probabilistic diffusion of a target object (or the aggregation after collapse) of mass *m* on the basis of the apparent diffusion rate (after deceleration) determined by the 1-particles forming it (before collapse); however, the distribution characteristics of the target object in its dispersion space are determined by the diffusion behavior of the 1-particles in the background field. When the particles are in the constrained state of ${\text {III}}u$, according to the above discussion, the case of $k>1$ must be considered. Then, the diffusion coefficient of a *k*-particle swarm can be obtained by substituting Eq. (32) into Eq. (31), namely,

33 $$\begin{aligned} D_k=-\frac{\hbar \, \Gamma ^2}{2\,m}\cdot \frac{1}{k}. \end{aligned}$$

This is equivalent to the proportional decline in the apparent diffusion rate of a target object (or the aggregation after collapse) of mass *m* due to the slowdown in the speed of the *k*-particles forming the target object. The meaning of the diffusion equation determined by this diffusion coefficient is similar to the case for 1-particles as considered above, that is, the dynamics of the probabilistic diffusion of a target object (or the aggregation after collapse) of mass *m* are described on the basis of the apparent diffusion rate (after deceleration) determined by the *k*-particles forming it (before collapse); however, the distribution characteristics of the target object in its dispersion space is determined by the diffusion behavior of the *k*-particles in the background field.

By taking the second partial derivative of $R(\varvec{{\mathcal {M}}}, k)$ (this is the plane vector sum in the boxes containing *k* moving particles, namely, the *k*-particle swarm, which is one of the component vectors in the entire microdomain ${\mathcal {V}}$) with respect to location (*x*, *y*, *z*), $\nabla ^2 R(\varvec{{\mathcal {M}}},k)$ can be obtained. It should be emphasized that the absolute sizes of the two (infinitesimal) microdomains ${\mathcal {V}}_{\textrm{A}}$ and ${\mathcal {V}}_{\textrm{B}}$, which are selected to compare their differences, are equal when calculating the derivative of the vector $\varvec{{\mathcal {M}}}$. After multiplying $\nabla ^2 R(\varvec{{\mathcal {M}}},k)$ by the diffusion coefficient for the *k*-particle swarm (Eq. 33) and then adding the products together from $k=1$ to $\infty$, the complete generalized diffusion expression (including coefficients) can be obtained as follows:

34 $$\begin{aligned} -\frac{\hbar \, \Gamma ^2}{2\,m}\sum _{k=1}^{\infty }\left[ \frac{1}{k}\cdot \nabla ^2 R\left( \varvec{{\mathcal {M}}},k\right) \right] . \end{aligned}$$

The diffusion calculated in this way is the generalized diffusion from the whole (infinitesimal) microdomain ${\mathcal {V}}_{\textrm{A}}$ to ${\mathcal {V}}_{\textrm{B}}$. Equation (34) can be simplified as follows:

35 $$\begin{aligned} -\frac{\hbar \, \Gamma ^2}{2\,m\,\mathrm e^{\varvec{{\mathcal {M}}}}}\left[ \nabla ^2\varvec{{\mathcal {M}}}-T^2(\varvec{{\mathcal {M}}})\right] , \end{aligned}$$

where $T^2(\varvec{{\mathcal {M}}})=\left( \dfrac{\partial \varvec{{\mathcal {M}}}}{\partial x}\right) ^{\!2}+\left( \dfrac{\partial \varvec{{\mathcal {M}}}}{\partial y}\right) ^{\!2}+\left( \dfrac{\partial \varvec{{\mathcal {M}}}}{\partial z}\right) ^{\!2}$. By combining the left-hand side of Eq. (8) with Eq. (35), a complete expression for the generalized diffusion equation for vectors is obtained:

36 $$\begin{aligned} {\textbf{i}}\,\frac{\partial \varvec{{\mathcal {M}}}}{\partial t}=-\frac{\hbar \, \Gamma ^2}{2\,m\,\mathrm e^{\varvec{{\mathcal {M}}}}}\left[ \nabla ^2\varvec{{\mathcal {M}}}-T^2(\varvec{{\mathcal {M}}})\right] . \end{aligned}$$

Therefore, the expression for the generalized diffusion coefficient with the two types of special constrained effects is given as37The diffusion coefficient here is not a constant but rather a natural exponential function that varies with the relative vector density of moving particles. Hence, the generalized diffusion equation and the generalized diffusion coefficient *Ɖ* for vectors in the constrained state of ${\text {III}}u$ have been determined. In this constrained state, the ratios of norms of the spatial equivalent vectors in a microdomain can be determined in accordance with the Poisson distribution, while the norms and directions of the spatial equivalent vectors in the complex plane can be determined in accordance with Eq. (36). Thus, the basic effective information for a spatial (moving) particle swarm in the constrained state of ${\text {III}}u$ has been derived.

The slowing down of diffusion based on spatial location is the only manifestation of the statistical effect of location aggregation (the second type of constrained state) in diffusion. Obviously, the second type of special constrained state effect of particles can be reflected according to the treatment method in Eq. (34). As mentioned above, the statistical effects include the location and direction aggregation in the constrained state of ${\text {III}}u$. For the case of velocity direction aggregation, because the particles are in the system with a speed of *u*, the diffusion coefficient will be affected by the $\Gamma$ effect, and the statistical effect of this case is also added to the equation. To brief, all of the statistical (constrained) effects in the constrained state of ${\text {III}}u$ have been incorporated into Eq. (34).

### Verification of Eq. (36)

**Figure 6:**
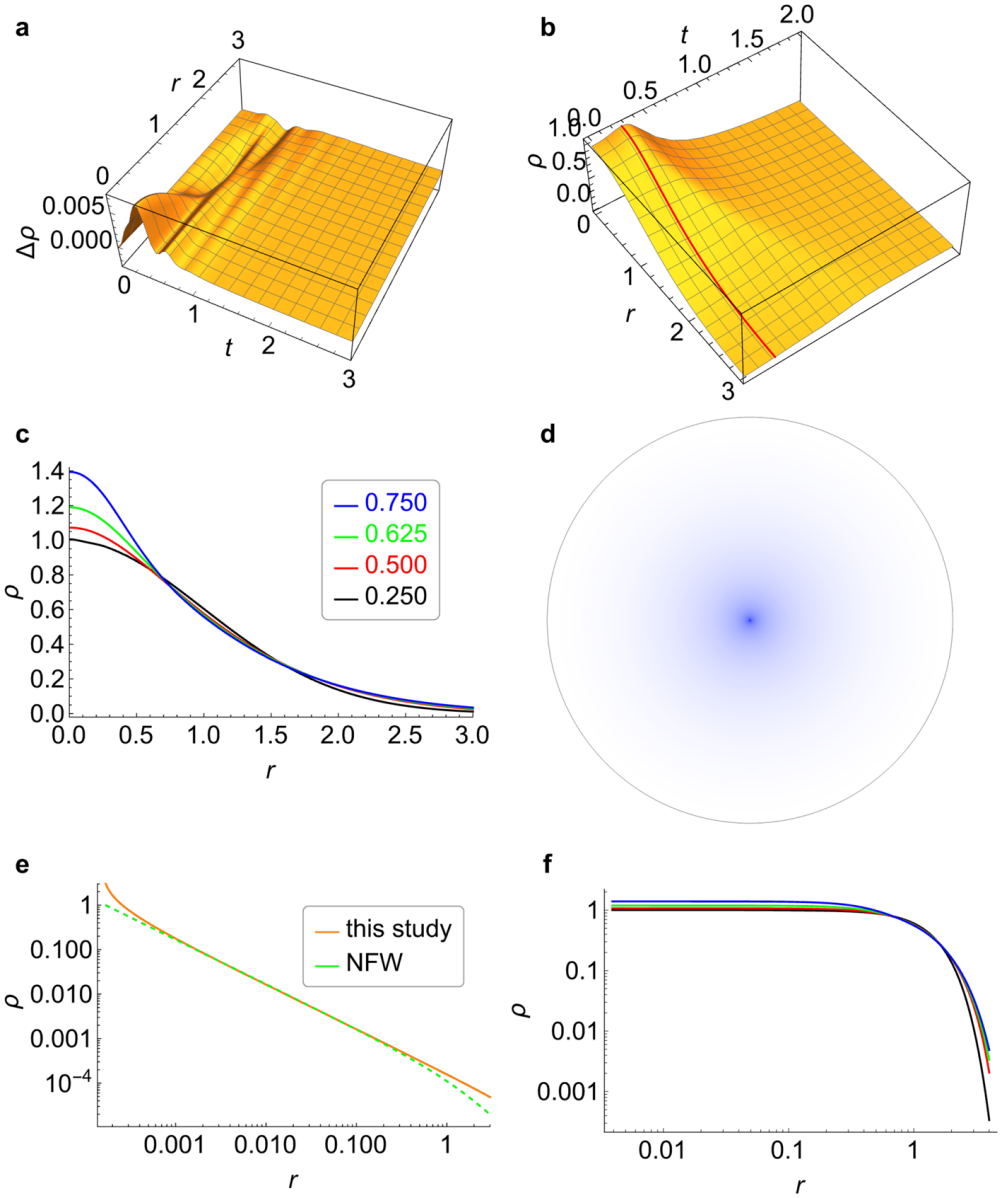
Prediction results ($\left\| \varvec{{\mathcal {M}}}\right\|$) of our equations in different cases. (**a**), Differences in density between the values calculated with Eq. (36) and the form of Schrödinger equation when the initial norm is $10^{-2}$. (**b**), Diffusion pattern of the Gaussian wave packet with time predicted by Eq. (36) when the initial norm is $\dfrac{1}{2}$. (**c**), Comparison of the radial distributions for different initial norms. (**d**), Radial distribution of the density (projected on the plane) integrated according to Eq. (40). (**e**), Comparison between the profiles of NFW and Eq. (40) ($r_{\textrm{c}}=\dfrac{1}{6000}$, $r_{\textrm{e}}=30$ and ${\mathcal {M}}_{\textrm{c}}=3+\,{\textbf{i}}$) on the scale of $r<r_{\textrm{s}}$. (**f**), Logarithmic profile of (**c**) as *r* varies from $0\sim 4$.

The derivation of Eq. (36) demonstrates that $\varvec{{\mathcal {M}}}$ is a relative vector, and the square of its first derivative is the higher-order infinitesimal of its second derivative. Also, the initial value of $\varvec{{\mathcal {M}}}$ could be a real, which is a relative density value relative to the T-domain. If the norm of the initial value (viz., the initial norm) is sufficiently small, Eq. (36) can be approximated as the form of Schrödinger equation without an external field when the $\Gamma$ effect is not considered. For example, while solving the diffusion problem of a 3-dimensional Gaussian wave packet formed by randomly moving particles, if the initial norm is less than $10^{-2}$, the solutions of the two equations are nearly identical (Fig. 6a, and the relative difference is less than $1\%$; note that the values of $\left\| \varvec{{\mathcal {M}}}\right\|$ which respect the mass density have been compared here). When the initial norm is sufficiently large, the particle swarm exhibits a certain degree of aggregation with time from the initial Gaussian wave packet. As shown in Fig. 6b, this aggregation is apparent at approximately $t=0.276$. As the initial norm increases, increasingly prominent aggregation processes appear. When the initial norms are 0.250, 0.500, 0.625 and 0.750, the radial distribution profile at the time of the most visible aggregation in each process (such as the red line in Fig. 6b) is taken to obtain the profile set, as shown in Fig. 6c (each profile is normalized according to the initial norm). It is speculated that when the initial norm increases to a certain value, a completely nondiffusive particle swarm may arise. Consequently, we have

38 $$\begin{aligned} \nabla ^2\varvec{{\mathcal {M}}}-T^2(\varvec{{\mathcal {M}}})=0, \end{aligned}$$

and $\varvec{{\mathcal {M}}}$ does not vary with time $t$ at this point. In the case of spherical symmetry, the boundary conditions of Eq. (38) can be given by

39 $$\begin{aligned} {\left\{ \begin{array}{ll} \varvec{{\mathcal {M}}}(r)={\mathcal {M}}_{\textrm{c}}, &{}r=r_{\textrm{c}},\\ \varvec{{\mathcal {M}}}(r)=0, &{}r=r_{\textrm{e}}, \end{array}\right. } \end{aligned}$$

where *r* is the distance to the spherical center; $r_{\textrm{c}}$ is the radius of inner boundary; $r_{\textrm{e}}$ is the radius of external boundary; ${\mathcal {M}}_{\textrm{c}}$ is a density constant. Then, the analytical solution can be obtained by solving the simultaneous equations of Eqs. 38 and 39:

40 $$\begin{aligned} \varvec{{\mathcal {M}}}(r)=\ln r-\ln \left[ \frac{r\left( r_{\textrm{c}}-r_{\textrm{e}}\,\mathrm e^{{\mathcal {M}}_{\textrm{c}}}\right) }{r_{\textrm{c}}r_{\textrm{e}}\left( \mathrm e^{{\mathcal {M}}_{\textrm{c}}}-1\right) }+1\right] +\ln \left[ \frac{\mathrm e^{{\mathcal {M}}_{\textrm{c}}}\left( r_{\textrm{c}}-r_{\textrm{e}}\right) }{r_{\textrm{c}}r_{\textrm{e}}\left( \mathrm e^{{\mathcal {M}}_{\textrm{c}}}-1\right) }\right] . \end{aligned}$$

See Part 3 of the Supplementary Information for the detailed Mathematica code of the solution process. Thus, given $r_{\textrm{c}}=\dfrac{1}{6000}$, $r_{\textrm{e}}=30$ and ${\mathcal {M}}_{\textrm{c}}=3+\,{\textbf{i}}$, the radial distribution of the mass density ($\left\| \varvec{{\mathcal {M}}}\right\|$) projected on the plane can be obtained, as illustrated in Fig. 6d.

For the universe, one of the scenarios corresponding to the particles in the constrained state of ${\text {III}}u$ is galaxies or galaxy clusters, which are affected only by gravitation. The results predicted by Eq. (40) are consistent with the observation results of relaxed galaxies and galaxy clusters (multiple images method). The NFW profile, as an empirical formula, is generally considered to be in good agreement with the observational results, which is given by

41 $$\begin{aligned} \rho (r)=\frac{\rho _{\textrm{c}}}{r/r_{\textrm{s}}(1+r/r_{\textrm{s}})^2}. \end{aligned}$$

Equation (41) shows that the shape of the profile is not affected by the parameters $\rho _{\textrm{c}}$ and $r_{\textrm{s}}$. The NFW profile was obtained by adjusting the two parameters, and the result was compared to the profile obtained with Eq. (40). The two profiles are almost consistent within the scale radius of $r_{\textrm{s}}$ (Fig. 6e). Therefore, Eq. (40) is in good agreement with the observational results of relaxed galaxy clusters within $r_{\textrm{s}}$, as mentioned in previous researches^11,12^; however, Eq. (40) is not consistent with the results in the range $r>r_{\textrm{s}}$. It is speculated that the inconsistency of these peripheral regions occurs because these galaxy clusters are not in completely nondiffusive states (diffusion is extremely slow when galaxy clusters are in these “relaxed” states because the principle masses are almost in nondiffusive states). The trend displayed in Fig. 6f shows that when the initial norm increases to a certain value, the radial distribution profiles of particle swarms diffusing from Gaussian wave packets in the range of $r>r_{\textrm{s}}$ are consistent with the observation results of the gravitational lens method. Furthermore, there are no cuspy problems emerging from Eq. (40). The central part of the particle swarm described by Eq. (40) can be a structure with a specific volume and a finite concentration. The peripheral distribution forms a stable “shell” to protect the central structure from diffusion.

Traditionally, the formation of such a mass distribution of relaxed galaxies or galaxy clusters is the result of gravitations. However, there is no interaction in the particles in the constrained state of ${\text {III}}u$ described in Eq. 40, which generates the same effect. A previous study^6^ proved that particles in the constrained state of ${\text {III}}u$ also experience the effects of special relativity. In addition, such particles can produce nondiffusive particle swarms of different scales. Accordingly, it is speculated that galaxies or galaxy clusters (at least dark matter halos) can be formed by these stochastic-constrained particles. In these constrained states, particles have fewer degrees of freedom in denser domains. And the apparent phenomenon of universal gravitation occurs between domains with fewer degrees of freedom and domains with more degrees of freedom. When randomly moving particles produce nondiffusive particle swarms, these particle swarms can also be regarded as particles with higher masses and can also produce larger nondiffusive particle swarms. Moreover, particles with higher masses move more slowly. If the universe is composed of such particles, at the micro level, there may be faster communication modes, and quantum entanglement and other fast communication phenomena are expected to be explained. At the macro level, the whole universe is fractal and the multi-body motion problems may be resolved by solving the generalized diffusion equation of their parent environment.

## Conclusions

Previous studies have focused on the overall behavior of randomly moving particle swarms. However, the characteristics of stochastic-constrained particle swarms that form ubiquitously in these swarms remain oblivious. In these special particle swarms, certain particular phenomena, such as the velocity or location aggregation effects, need to be considered. Although general relativity describes the influence of mass on space-time or motion, it does not give a complete diffusion equation. This study demonstrated a generalized diffusion equation for randomly moving particles in the constrained state of ${\text {III}}u$ observed from their parent particle swarm. When the norm of the initial value is small, the equation can be approximated as the form of Schrödinger equation; when the norm is large, the equation can be used to describe the aggregation process of particles. Although our model describes a noninteracting particle swarm, it encompasses the apparent phenomena of universal gravitation.

In the more general case, i.e., in the third type of general constrained state, we can divide the whole system into countless fragments according to the time and domain. Each fragment can be approximated as in the constrained state of ${\text {III}}u$. We utilize Eq. (36) to determine the results for each segment and splice them together. Thus, the whole problem of the third type of general constraint can be solved.

## Supplementary Information

Supplementary Information.

## Data Availability

All data generated or analysed during this study are included in this published article and its supplementary information files.
