# Supplementary material for: Dynamics of stochastic-constrained particles
Source: Sci Rep. 2023 Feb 16;13:2759. doi: 10.1038/s41598-023-29940-y (PMC9935625; doi:10.1038/s41598-023-29940-y)
Supplement: Supplementary file 1 — Supplementary Information. [file 41598_2023_29940_MOESM1_ESM.pdf]

Appendix:

Supplementary Information
(Mathematica v13.1.0 code of TraditionalForm)

Dynamics of Stochastic-constrained Particles

Tao Guo

Center for Drug Delivery System, Shanghai Institute of Materia Medica, Chinese Academy of Sciences, 501 Haike Road, Shanghai 201210, China. E-mail: guotao@simm.ac.cn; gotallcn@gmail.com

NOTE:
1. The "Euclid Math One" regular and bold fonts are needed to display the contents correctly in this Notebook.
2. If there is no special case, the Mathematica code starts with gray "*In*[∘]:=" and is bold by default according to Mathematica's rules.

Part 1. The Norm of the Component Vector is Proportional to the Number of Vectors Forming It

When the total vector value of a specified vector swarm is determined, the mean norms between different component vectors should be proportional to the number forming them in the constrained state of IIIu. The following proves this viewpoint in detail.

According to my previous study[1], let $\mathcal{M}$k being the norm of momentum of k particles observed from $\mathcal{R}_u$, the probability density of momentum norm formed by k particles in $\mathcal{R}_u$ observed in $\mathcal{R}_0$ can be expressed as (This code takes approximately 71 seconds):

In[∘]:=
```
Clear["Global`*"];
𝒟 = TransformedDistribution[ Sqrt[(k u)^2 + ℳk^2 - 2 k u ℳk Cos[ArcCos[η]]] ,
    {ℳk ≈ MaxwellDistribution[ (Sqrt[k] Sqrt[c^2 - u^2]) / Sqrt[3] ], η ≈ UniformDistribution[{-1, 1}]}];
FullSimplify[PDF[𝒟, x], Assumptions → c > u > 0 ∧ k > 0]
```

Out[∘]=

$$\begin{cases} \dfrac{\sqrt{3}\, x \left(e^{\frac{6 u x}{c^2-u^2}}-1\right) e^{-\frac{3 (k u+x)^2}{2 k \left(c^2-u^2\right)}}}{k u \sqrt{2 \pi c^2 k-2 \pi k u^2}} & (x>0 \land k u>x) \lor k u<x \\ -\dfrac{\sqrt{6 \pi} \sqrt{c^2 k-u x} \left(5 u x-2 c^2 k\right) \operatorname{erf}\left(\frac{\sqrt{6}\, x}{\sqrt{c^2 k-u x}}\right)+4 x e^{\frac{6 x^2}{u x-c^2 k}} \left(c^2 (6 k+2)-u (2 u+3 x)\right)-8 x (c-u) (c+u)}{4 \sqrt{6 \pi}\, k^{5/2} u ((c-u) (c+u))^{3/2}} & k u=x \end{cases}$$

The first branch is selected as valid.

In view of the above conclusions, we find the mean value of this distribution (This code takes approximately 31 seconds).

In[∘]:=
```
𝒴̅_k = FullSimplify[
    Mean[ProbabilityDistribution[ (Sqrt[3] x (e^((6 u x)/(c^2 - u^2)) - 1) e^(-(3 (k u + x)^2)/(2 k (c^2 - u^2)))) / (k u Sqrt[2 π c^2 k - 2 π k u^2]), {x, 0, +∞}]], Assumptions → c > u > 0 ∧ k > 0]
```

Out[∘]=

$$\frac{\left(c^2+(3 k-1) u^2\right) \operatorname{erf}\left(\frac{\sqrt{\frac{3}{2}}\, k u}{\sqrt{k (c-u) (c+u)}}\right)+\sqrt{\frac{6}{\pi}}\, u\, e^{\frac{3 k u^2}{2\left(u^2-c^2\right)}} \sqrt{k (c-u) (c+u)}}{3 u}$$

We find the limit of the ratio of this mean value $\overline{\mathcal{Y}}_k$ and k when k approaches $+\infty$.

In[ ]:= **Simplify[Limit[$\frac{\overline{\mathcal{Y}}_k}{k}$, $k \to +\infty$], Assumptions → $u > 0$]**

Out[ ]=

$$\begin{cases} -u & \arg(c^2 - u^2) \geq \pi \\ u & \text{True} \end{cases}$$

The second brunch is meaningful. Therefore, when k is a large number, the norm of the mean value $\overline{\boldsymbol{\mathcal{Y}}}_k$ is directly proportional to the number k forming $\overline{\boldsymbol{\mathcal{Y}}}_k$, namely, $\overline{\mathcal{Y}}_k = k \cdot u$.

Eq. 11 in the main text determines the proportion of particle number distributed in various boxes partitioned by k, and these particles are distributed in each box of $\mathcal{V}$ with equal probability. That is, the particles are randomly extracted from the microdomain $\mathcal{V}$ to be distributed in each box. When the number of extractions is large enough, the norm of each component vector partitioned by k should be directly proportional to the number of particles according to the probability and the scale factor is u.

The unique expansion of scalar $\mathcal{M}$ in the form of including power series is

$$\mathcal{M} = \sum_{k=1}^{\infty} \frac{e^{-\mathcal{M}} \mathcal{M}^k}{(k-1)!}$$

If the corresponding terms marked by k are directly proportional between the expansion of the norm $\|\boldsymbol{\mathcal{M}}\|$ of vector $\boldsymbol{\mathcal{M}}$ and the expansion of the scalar $\mathcal{M}$ representing the number of particles, or the numbers of particles are allowed to be proportional to the norms of vectors they form, the number $\mathcal{M}$ of particles must be equal to the norm $\|\boldsymbol{\mathcal{M}}\|$ of the vector $\boldsymbol{\mathcal{M}}$ they form besides they are required to obey Poisson distribution. According to the above conclusion $\overline{\mathcal{Y}}_k = k \cdot u$, the average speed $u = 1$ is needed in the system.

References

[1] Guo, T. Study on the average speed of particles from a particle swarm derived from a stationary particle swarm. *Scientific Reports* **11**, 1–4 (2021).

Part 2. The Square of the Norm of the Average Velocity is Proportional to the Number of Vectors

As described in the main text, the k-particle is a general particle composed of k 1-particles. Each 1-particle is moving at the same speed c and in a random direction in the 3-dimensional Cartesian coordinate system (they are in a completely free state or in the constrained state of Iu not considering the Γ effect). Suppose that the standard deviation of the projection of the velocity of any one of the k equivalent 1-particles forming a k-particle onto each equivalent coordinate axis is σ. According to the my previous study[1], the speed of k-particles (or k particles in a certain domain) follows the Maxwell distribution with scale parameter $\frac{\sigma}{\sqrt{k}}$.

Then, the average velocity of the k-particles (or k particles in a certain domain) is

In[ ]:= **$\bar{v}$ = Mean[MaxwellDistribution[$\frac{\sigma}{\sqrt{k}}$]]**

Out[ ]=

$$\frac{2\sqrt{\frac{2}{\pi}}\,\sigma}{\sqrt{k}}$$

For k_a- and k_b-particles, the ratio of their average velocity $\bar{v}_a / \bar{v}_b$ =

In[∘]:= $$\frac{2\sqrt{\frac{2}{\pi}}\,\sigma}{\sqrt{k_{\mathrm{a}}}} \Big/ \frac{2\sqrt{\frac{2}{\pi}}\,\sigma}{\sqrt{k_{\mathrm{b}}}}$$

Out[∘]=

$$\frac{\sqrt{k_b}}{\sqrt{k_a}}$$

And because: $m_{\mathrm{a}} = \mu\, k_{\mathrm{a}}$ and $m_{\mathrm{b}} = \mu\, k_{\mathrm{b}}$, where μ is the scale factor or the mass of 1-particle. $\bar{v}_{\mathrm{a}} / \bar{v}_{\mathrm{b}}$ is also equal to

In[∘]:= $$\mathbf{Simplify}\left[\frac{\sqrt{\frac{m_{\mathrm{b}}}{\mu}}}{\sqrt{\frac{m_{\mathrm{a}}}{\mu}}}, \mathbf{Assumptions} \to \mu > 0\right]$$

Out[∘]=

$$\frac{\sqrt{m_b}}{\sqrt{m_a}}$$

Therefore, the square of the average velocity of particles is directly proportional to the mass of particles or the number of 1-particles forming it.

References

[1] Guo, T. Study on the average speed of particles from a particle swarm derived from a stationary particle swarm. *Scientific Reports* **11**, 1–4 (2021).

Part 3. Solving Process of Eq. 38 in the Main Text

To solve the partial differential equation Eq. 38 in the main text, it is assumed that the system is spherically symmetric because it is isotropic at a huge scale. Therefore, we make the conversion from rectangular to spherical coordinates (note that φ is used to denote the azimuthal angle, whereas θ is used to denote the polar angle), namely, $x = r \sin\theta \cos\varphi$, $y = r \sin\theta \sin\varphi$ and $z = \cos\theta$.

In the case of spherical symmetry, the change of function $\mathcal{M}(r)$ does not depend on θ and φ, but is related to r. Therefore, after the coordinate transformation, and the first and the second derivatives are obtained, to omit the terms that depends on angles θ and φ, we can obtain (subject to the character limitation of Mathematica, $\mathcal{M}$ is used instead of $\mathcal{M}$ in the code cell; the same is done below):

In[∘]:= $$\mathbf{Simplify}\Big[\frac{2}{r} D[\mathcal{M}[r], \{r, 1\}] + D[\mathcal{M}[r], \{r, 2\}] - (D[\mathcal{M}[r], \{r, 1\}])^2 \left((\mathrm{Sin}[\theta]\,\mathrm{Cos}[\varphi])^2 + (\mathrm{Sin}[\theta]\,\mathrm{Sin}[\varphi])^2 + (\mathrm{Cos}[\theta])^2\right)\Big]$$

Out[∘]=

$$\mathcal{M}''(r) - \mathcal{M}'(r)^2 + \frac{2\,\mathcal{M}'(r)}{r}$$

To solve the abovementioned differential equation under the boundary condition $\mathcal{M}(r_{\mathrm{e}}) = 0$.

In[∘]:= $$\mathbf{DSolve}\left[\left\{\mathcal{M}''[r] - (\mathcal{M}'[r])^2 + \frac{2}{r}\mathcal{M}'[r] == 0, \mathcal{M}[\mathrm{re}] == 0\right\}, \mathcal{M}[r], r\right]$$

Out[∘]=

$$\{\{\mathcal{M}(r) \to \log(r) - \log(1 + c_1\, r) - \log(\mathrm{re}) + \log(1 + c_1\, \mathrm{re})\}\}$$

Suppose another boundary condition is $\mathcal{M}(r_{\mathrm{c}}) = \mathcal{M}_{\mathrm{c}}$, then

In[ ]:= $r = \text{rc};$
Solve[Log[r] − Log[1 + c1 r] − Log[re] + Log[1 + c1 re] == $\mathcal{M}$c, c1]

Out[ ]=

$$\left\{\left\{\text{c1} \to \frac{\text{rc} - \text{re}\, e^{\mathcal{M}\text{c}}}{\text{rc re}\left(e^{\mathcal{M}\text{c}} - 1\right)}\right\}\right\}$$

Therefore, the solution of the above differential equation is as follows:

In[ ]:= Clear["Global`*"];

$$\text{c1} = \frac{\text{rc} - \text{re}\, e^{\mathcal{M}\text{c}}}{\text{rc re}\left(e^{\mathcal{M}\text{c}} - 1\right)};$$

Simplify[Log[r] − Log[1 + c1 r] − Log[re] + Log[1 + c1 re]]

Out[ ]=

$$-\log\left(\frac{r\left(\text{rc} - \text{re}\, e^{\mathcal{M}\text{c}}\right)}{\text{rc re}\left(e^{\mathcal{M}\text{c}} - 1\right)} + 1\right) + \log(r) + \log\left(\frac{e^{\mathcal{M}\text{c}}(\text{rc} - \text{re})}{\text{rc}\left(e^{\mathcal{M}\text{c}} - 1\right)}\right) - \log(\text{re})$$

To restore the above solution in spherical to the solution in 3-dimensional rectangular coordinates, then

In[ ]:= $r = \sqrt{x^2 + y^2 + z^2};$

$$\text{FullSimplify}\left[-\text{Log}\left[\frac{r\left(\text{rc} - \text{re}\, e^{\mathcal{M}\text{c}}\right)}{\text{rc re}\left(e^{\mathcal{M}\text{c}} - 1\right)} + 1\right] + \text{Log}[r] + \text{Log}\left[\frac{e^{\mathcal{M}\text{c}}(\text{rc} - \text{re})}{\text{rc}\left(e^{\mathcal{M}\text{c}} - 1\right)}\right] - \text{Log}[\text{re}],\right.$$
$$\left.\text{Assumptions} \to \text{re} > \text{rc} > 0\right]$$

Out[ ]=

$$-\log\left(\frac{\left(\text{rc} - \text{re}\, e^{\mathcal{M}\text{c}}\right)\sqrt{x^2 + y^2 + z^2}}{e^{\mathcal{M}\text{c}} - 1} + \text{rc re}\right) + \log\left(\frac{e^{\mathcal{M}\text{c}}(\text{rc} - \text{re})}{e^{\mathcal{M}\text{c}} - 1}\right) + \frac{1}{2}\log\left(x^2 + y^2 + z^2\right)$$

To verify the above results:

In[ ]:=

$$\mathcal{M}[x, y, z] := -\text{Log}\left[\frac{\left(c - \text{re}\, e^{\mathcal{M}\text{c}}\right)\sqrt{x^2 + y^2 + z^2}}{e^{\mathcal{M}\text{c}} - 1} + \text{rc re}\right] + \text{Log}\left[\frac{e^{\mathcal{M}\text{c}}(\text{rc} - \text{re})}{e^{\mathcal{M}\text{c}} - 1}\right] + \frac{1}{2}\text{Log}\left[x^2 + y^2 + z^2\right];$$

$$\text{FullSimplify}\left[\frac{\partial^2 \mathcal{M}(x, y, z)}{\partial x^2} + \frac{\partial^2 \mathcal{M}(x, y, z)}{\partial y^2} + \frac{\partial^2 \mathcal{M}(x, y, z)}{\partial z^2} - \left(\frac{\partial \mathcal{M}(x, y, z)}{\partial x}\right)^2 - \left(\frac{\partial \mathcal{M}(x, y, z)}{\partial y}\right)^2 - \left(\frac{\partial \mathcal{M}(x, y, z)}{\partial z}\right)^2\right]$$

Out[ ]=

0

Therefore, the above equation is the solution of Eq. 38 in the main text (only when Im[$\mathcal{M}_0$] $\in [-\pi, \pi)$ and the principal values of arguments are taken in the calculation process).

Similarly, the 2-dimensional case can also be solved.

In[ ]:= Clear["Global`*"];

$$\text{Simplify}\left[D[\mathcal{M}[r], \{r, 2\}] + \frac{1}{r} D[\mathcal{M}[r], \{r, 1\}] - (D[\mathcal{M}[r], \{r, 1\}])^2\right]$$

Out[ ]=

$$\mathcal{M}''(r) - \mathcal{M}'(r)^2 + \frac{\mathcal{M}'(r)}{r}$$

In[ ]:= $\mathbf{DSolve}\left[\left\{\mathcal{M}''[r]-\mathcal{M}'[r]^2+\frac{\mathcal{M}'[r]}{r}==0,\ \mathcal{M}[\mathrm{re}]==0\right\},\ \mathcal{M}[r],\ r\right]$

Out[ ]=

$\{\{\mathcal{M}(r)\to\log(-\log(\mathrm{re})+c_1)-\log(-\log(r)+c_1)\}\}$

In[ ]:= $r=\mathrm{rc};$

Solve[Log[−Log[re] + c1] − Log[−Log[*r*] + c1] == $\mathcal{M}$c, c1]

Out[ ]=

$\left\{\left\{\mathrm{c1}\to\frac{e^{\mathcal{M}\mathrm{c}}\log(\mathrm{rc})-\log(\mathrm{re})}{e^{\mathcal{M}\mathrm{c}}-1}\right\}\right\}$

In[ ]:= **Clear["Global`*"];**

$\mathbf{c1}=\frac{e^{\mathcal{M}\mathrm{c}}\,\mathbf{Log[rc]-Log[re]}}{e^{\mathcal{M}\mathrm{c}}-1};$

Simplify[Log[−Log[re] + c1] − Log[−Log[*r*] + c1]]

Out[ ]=

$\log\left(\frac{e^{\mathcal{M}\mathrm{c}}(\log(\mathrm{rc})-\log(\mathrm{re}))}{e^{\mathcal{M}\mathrm{c}}-1}\right)-\log\left(\frac{e^{\mathcal{M}\mathrm{c}}\log(\mathrm{rc})-\log(\mathrm{re})}{e^{\mathcal{M}\mathrm{c}}-1}-\log(r)\right)$

In[ ]:= $r=\sqrt{x^2+y^2};$

$\mathbf{FullSimplify}\left[\mathrm{Log}\left[\frac{e^{\mathcal{M}\mathrm{c}}(\mathrm{Log[rc]}-\mathrm{Log[re]})}{e^{\mathcal{M}\mathrm{c}}-1}\right]-\mathrm{Log}\left[\frac{e^{\mathcal{M}\mathrm{c}}\,\mathrm{Log[rc]}-\mathrm{Log[re]}}{e^{\mathcal{M}\mathrm{c}}-1}-\mathrm{Log}[r]\right],\right.$

$\left.\mathrm{Assumptions}\to\mathrm{re}>\mathrm{rc}>0\right]$

Out[ ]=

$\log\left(\frac{e^{\mathcal{M}\mathrm{c}}\log\left(\frac{\mathrm{rc}}{\mathrm{re}}\right)}{e^{\mathcal{M}\mathrm{c}}-1}\right)-\log\left(\frac{\log\left(\frac{\mathrm{rc}}{\mathrm{re}}\right)}{e^{\mathcal{M}\mathrm{c}}-1}+\log(\mathrm{rc})-\frac{1}{2}\log\left(x^2+y^2\right)\right)$

In[ ]:= $\mathcal{M}[x,y]:=\mathrm{Log}\left[\frac{e^{\mathcal{M}\mathrm{c}}\,\mathrm{Log}\left[\frac{\mathrm{rc}}{\mathrm{re}}\right]}{e^{\mathcal{M}\mathrm{c}}-1}\right]-\mathrm{Log}\left[\frac{\mathrm{Log}\left[\frac{\mathrm{rc}}{\mathrm{re}}\right]}{e^{\mathcal{M}\mathrm{c}}-1}+\mathrm{Log[rc]}-\frac{1}{2}\mathrm{Log}\left[x^2+y^2\right]\right];$

$\mathbf{FullSimplify}\left[\frac{\partial^2\mathcal{M}(x,y)}{\partial x^2}+\frac{\partial^2\mathcal{M}(x,y)}{\partial y^2}-\left(\frac{\partial\mathcal{M}(x,y)}{\partial x}\right)^2-\left(\frac{\partial\mathcal{M}(x,y)}{\partial y}\right)^2\right]$

Out[ ]=

0

To verify the above conclusion, the results of analytical solution and the numerical solution under the same conditions are plotted (This code takes approximately 32 seconds):

In[ ]:= **Clear["Global`*"];**

$\mathcal{M}\mathrm{a}[\mathrm{x\_},\mathrm{y\_}]:=\mathrm{Log}\left[\frac{e^{\mathcal{M}\mathrm{c}}\,\mathrm{Log}\left[\frac{\mathrm{rc}}{\mathrm{re}}\right]}{e^{\mathcal{M}\mathrm{c}}-1}\right]-\mathrm{Log}\left[\frac{\mathrm{Log}\left[\frac{\mathrm{rc}}{\mathrm{re}}\right]}{e^{\mathcal{M}\mathrm{c}}-1}+\mathrm{Log[rc]}-\frac{1}{2}\mathrm{Log}\left[x^2+y^2\right]\right];$

$\mathrm{rc}=\frac{4}{100};$

re = 4;

$\mathcal{M}\mathrm{c}=1+2\,i;$

$\Omega=\mathrm{ImplicitRegion}\left[\mathrm{rc}^2\le x^2+y^2\le\mathrm{re}^2,\ \{x,y\}\right];$

$\mathrm{G1}=\mathrm{Show}\left[\mathrm{Plot3D}\left[\mathrm{Norm}[\mathcal{M}\mathrm{a}[x,y]],\ \{x,y\}\in\Omega,\ \mathrm{PlotRange}\to\left\{0,\sqrt{8}\right\},\right.\right.$

ColorFunction → (Hue[0.65, #3] &), MeshStyle → None, BoundaryStyle → None, PlotPoints → 300,

$\mathrm{AxesLabel}\to\left\{\mathrm{Style}[\text{"}x\ \text{", Italic}],\ \mathrm{Style}[\text{"y", Italic}],\ \mathrm{Rotate}\left[\mathrm{Style}[\text{"Density }\quad\text{"}],\ \frac{\pi}{2}\right]\right\},$

```
    AxesStyle → Directive[Black, FontFamily → "Arial", FontSize → 15], TicksStyle → Black,
    BoxStyle → Directive[Black, Thickness → 0.0018], BoxRatios → Automatic, ViewPoint → {15, −26, 16},
    Epilog → Text[Style["a", 15, FontFamily → "Arial", Bold, Black], {−0.07, 0.92}, {−1, 1}]],
  Table[Ω1 = ImplicitRegion[9/100 ≤ x^2 + i^2 ≤ 16, {x}]; If[i^2 ≤ 9/100, xx = Sqrt[9/100 − i^2], xx = 0];
    ParametricPlot3D[{x, i, Norm[Ma[x, i]]}, {x} ∈ Ω1, PlotStyle → Thickness[0.0018], PlotPoints → 300,
      ColorFunction → (GrayLevel[0.4, 1 − #3 × Norm[Ma[xx, i]]/Norm[Ma[0, 3/10]]] &)], {i, −3.5, 3.5, 0.5}],
  Table[Ω1 = ImplicitRegion[9/100 ≤ j^2 + y^2 ≤ 16, {y}]; If[j^2 ≤ 9/100, yy = Sqrt[9/100 − j^2], yy = 0];
    ParametricPlot3D[{j, y, Norm[Ma[j, y]]}, {y} ∈ Ω1, PlotStyle → Thickness[0.0018],
      PlotPoints → 300, ColorFunction → (GrayLevel[0.4, 1 − #3 × Norm[Ma[j, yy]]/Norm[Ma[0, 3/10]]] &)],
    {j, −3.5, 3.5, 0.5}], ParametricPlot3D[{4 Cos[ϕ], 4 Sin[ϕ], 0}, {ϕ, 0, 2 π},
    PlotStyle → Directive[Gray, Thickness[0.0018]], PlotPoints → 300]];
Needs["NDSolve`FEM`"];
mesh = ToElementMesh[Ω, MeshRefinementFunction →
    Function[{vertices, area}, area > 3/100000 (1/10 + 80 Norm[Mean[vertices]])]];
Mn = NDSolveValue[{D[u[x, y], {x, 2}] + D[u[x, y], {y, 2}] − (D[u[x, y], x])^2 − (D[u[x, y], y])^2 == 0, DirichletCondition[
     u[x, y] == Mc, x^2 + y^2 == rc^2], DirichletCondition[u[x, y] == 0, x^2 + y^2 == re^2]}, u, {x, y} ∈ mesh];
G2 = Show[Plot3D[Norm[Mn[x, y]], {x, y} ∈ mesh, PlotRange → {0, Sqrt[8]},
    ColorFunction → (Hue[0.65, #3] &), MeshStyle → None, BoundaryStyle → None,
    AxesLabel → {Style["x  ", Italic], Style["y", Italic], Rotate[Style["Density     "], π/2]},
    AxesStyle → Directive[Black, FontFamily → "Arial", FontSize → 15], TicksStyle → Black,
    BoxStyle → Directive[Black, Thickness → 0.002], BoxRatios → Automatic, ViewPoint → {15, −26, 16},
    Epilog → Text[Style["b", 15, FontFamily → "Arial", Bold, Black], {−0.07, 0.92}, {−1, 1}]],
  Table[Ω2 = ImplicitRegion[9/100 ≤ x^2 + i^2 ≤ 16, {x}]; If[i^2 ≤ 9/100, xx = Sqrt[9/100 − i^2], xx = 0];
    ParametricPlot3D[{x, i, Norm[Mn[x, i]]}, {x} ∈ Ω2, PlotStyle → Thickness[0.0018], PlotPoints → 300,
      ColorFunction → (GrayLevel[0.4, 1 − #3 × Norm[Mn[xx, i]]/Norm[Mn[0, 3/10]]] &)], {i, −3.5, 3.5, 0.5}],
  Table[Ω2 = ImplicitRegion[9/100 ≤ j^2 + y^2 ≤ 16, {y}]; If[j^2 ≤ 9/100, yy = Sqrt[9/100 − j^2], yy = 0];
    ParametricPlot3D[{j, y, Norm[Mn[j, y]]}, {y} ∈ Ω2, PlotStyle → Thickness[0.0018],
```

```
PlotPoints → 300, ColorFunction → (GrayLevel[0.4, 1 − #3 × Norm[𝓜n[j, yy]] / Norm[𝓜n[0, 3/10]]] &)],
  {j, −3.5, 3.5, 0.5}], ParametricPlot3D[{4 Cos[ϕ], 4 Sin[ϕ], 0}, {ϕ, 0, 2 π},
  PlotStyle → Directive[Gray, Thickness[0.0018]], PlotPoints → 300]];
s1 = GraphicsRow[{G1, G2}, ImageSize → 500, Spacings → Scaled[−0.06]];
Pane[s1, {500, 200}, ImageMargins → {{50, −30}, {−18, −25}}]
```

Out[∘]=

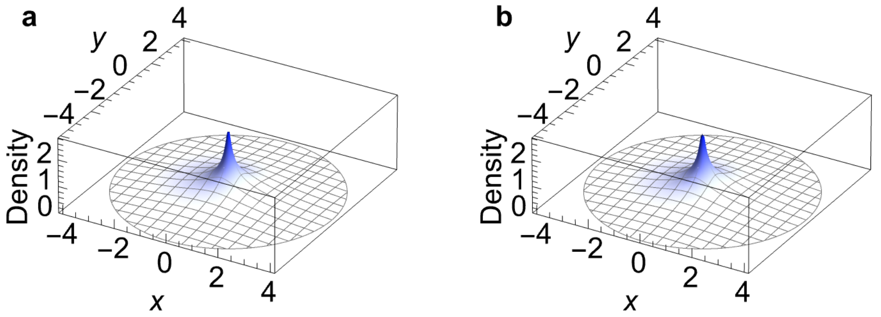

Figure S1 | Distribution of the mass density of a particle swarm meeting conditions ($\mathcal{M}(x, y) = 1 + 2i \wedge x^2 + y^2 = \frac{16}{10000}$) $\wedge$ ($\mathcal{M}(x, y) = 0 \wedge x^2 + y^2 = 4^2$). **a**, The analytical solution. **b**, The numerical solution.

It can be seen from Fig. S1b that the numerical solution and the analytical solution achieve a perfect agreement (only when $\text{Im}[\mathcal{M}_c] \in [-\pi, \pi)$ and the principal values of arguments are taken in the calculation process).

Part 4. Figures Used in the Main Text

NOTE: To run these codes correctly, the contents in "**MyDirection = ∗∗**" in the next cell should be modified. It is similar to **MyDirection** = "**/Users/yourdirection/**". Then, run it (Shift+Enter) beforehand.

```
MyDirection = ∗∗;
Protect[MyDirection];
Off[General::wrsym];
```

```
## ## ## ## ## ## ## ## ## ## ## Figure1 ## ## ## ## ## ## ## ## ## ## ## ## ## ## ##
```

```
In[ ]:= Clear["Global`*"];
head = Graphics[Polygon[0.13*{{-1, 8.09/25}, {0, 0}, {-1, -8.09/25}, {-8.09/10, 0}, {-1, 8.09/25}}]];
aa = Graphics[{{Blue, Thickness[0.003], Circle[{0, 1/2}, 1.04]},
    {Red, Thickness[0.003], Circle[{0, 0}, 2]}, {RGBColor[0, 0, 1, 1],
      Arrowheads[{{.3, 1, {head, 0.06}}}], {Thickness[0.006], Arrow[{{0, 0.5}, {1.4, 0.5}}]}},
    {Green, PointSize[0.01], Point[{0, 1/5}]},
    Text[Style["R", 18, FontFamily -> "Euclid Math One", Blue], {0, 1.07}],
    Text[Style["u", Italic, 12, FontFamily -> "Arial", Blue], {0.132, 1.03}],
    Text[Style["Target (Sub-) domain", 18, FontFamily -> "Arial", Blue], {0.01, 2/3}],
    Text[Style["Total (Parent/Background) domain", 18, FontFamily -> "Arial", Red], {0, -4/5}],
    Text[Style["R", 18, FontFamily -> "Euclid Math One", Red], {0, -1.25}],
    Text[Style["0", 12, FontFamily -> "Arial", Red], {0.132, -1.3}],
    Text[Style["Microdomain", 18, FontFamily -> "Arial", Green], {0, 0}],
    Text[Style["u", Italic, 18, FontFamily -> "Arial", Blue], {1.53, 0.51}]}];
Export[MyDirection <> "figure1.eps", aa, Background -> None];

## ## ## ## ## ## ## ## ## ## ## Figure1 ## ## ## ## ## ## ## ## ## ## ## ## ## ## ##

## ## ## ## ## ## ## ## ## ## ## Figure2 ## ## ## ## ## ## ## ## ## ## ## ## ## ## ##

In[ ]:= Clear["Global`*"];
{rr1, bb1} = Last@Reap@
    Scan[If[#[[1]]^2 + #[[2]]^2 < 1, Sow[#, "Red"], Sow[#, "Blue"]] &, RandomReal[{-2, 2}, {2000, 2}]];
𝓡1 = ImplicitRegion[x^2 + y^2 > 1, {{x, -2, 2}, {y, -2, 2}}];
𝓡2 = ImplicitRegion[x^2 + y^2 < 1, {{x, -2, 2}, {y, -2, 2}}];
{rr2, bb2} = {RandomPoint[𝓡1, 1000], RandomPoint[𝓡2, 600]};
head = Graphics[Polygon[0.1*{{-1, 8.09/25}, {0, 0}, {-1, -8.09/25}, {-8.09/10, 0}, {-1, 8.09/25}}]];
bb = Graphics[{{Blue, Dashed, Thickness[0.0016], Circle[{0, 0}, 1]}, {Red, Point[rr1]}, {Blue, Point[bb1]},
    {Blue, Dashed, Thickness[0.0016], Circle[{4.5, 0}, 1]}, {RGBColor[0, 0, 1, 1],
      Arrowheads[{{0.2, 1, {head, 0.03}}}], {Thickness[0.004], Arrow[{{0, 0}, {1.37, 0}}]}},
    Text[Style["u", 20, Italic, FontFamily -> "Arial", Blue], {1.53, 0.01}],
    Text[Style["a", 20, Bold, FontFamily -> "Arial", Black], {-2, 2}],
    {Red, Point[rr2 + Table[{4.5, 0}, {i, Length[rr2]}]]},
    {Blue, Point[bb2 + Table[{4.5, 0}, {i, Length[bb2]}]]},
    {Blue, Arrowheads[{{.2, 1, {head, 0.03}}}], {Thickness[0.004], Arrow[{{4.5, 0}, {5.87, 0}}]}},
    Text[Style["u", 20, Italic, FontFamily -> "Arial", Blue], {6.03, 0.01}],
    Text[Style["b", 20, Bold, FontFamily -> "Arial", Black], {2.5, 2}]},
   Epilog -> Inset[LineLegend[{Directive[Blue, Thickness[0.004]], Directive[Red, Thickness[0.004]]},
      {Style["Particles included in statistics", FontFamily -> "Arial", FontSize -> 20],
        Style["Particles not included in statistics", FontFamily -> "Arial", FontSize -> 20]},
      Joined -> {False, False}, LegendLayout -> "Row", LegendFunction ->
        (Framed[#, RoundingRadius -> 4, Background -> White, FrameStyle -> GrayLevel[0.58]] &)],
     Scaled[{1/2, 0.11}]], ImageSize -> 700];
Export[MyDirection <> "figure2.eps", bb, Background -> None];
```

```
## ## ## ## ## ## ## ## ## ## ## Figure2 ## ## ## ## ## ## ## ## ## ## ## ## ## ## ##

## ## ## ## ## ## ## ## ## ## ## Figure3 ## ## ## ## ## ## ## ## ## ## ## ## ## ## ##

In[ ]:= Clear["Global`*"];
head = Graphics[Polygon[0.3*{{-1, 8.09/25}, {0, 0}, {-1, -8.09/25}, {-8.09/10, 0}, {-1, 8.09/25}}]];
cc = Graphics[{{RGBColor[178/255, 252/255, 61/255], Rectangle[{0, 0}, {1, 1}]},
    {RGBColor[178/255, 252/255, 61/255, 0.5], Rectangle[{1, 0}, {2, 1}]}, {RGBColor[250/255, 200/255, 0],
      Arrowheads[{{0.2, 1, {head, 0.06}}}], {Thickness[0.006], Arrow[{{0.7, 0.54}, {1.3, 0.54}}]}},
    {RGBColor[250/255, 200/255, 0], Arrowheads[{{0.2, 1, {head, 0.06}}}],
      {Thickness[0.006], Arrow[{{1.3, 0.46}, {0.7, 0.46}}]}}, {RGBColor[178/255, 252/255, 61/255, 0.5],
      Arrowheads[0.06], {Thickness[0.006], Arrow[{{0, -1.3}, {1.2, -1.3}}]}},
    {RGBColor[178/255, 252/255, 61/255], Arrowheads[0.06], {Thickness[0.006], Arrow[{{0, -1.3}, {0.8, -0.3}}]}},
    {Orange, {Thickness[0.0036], DotDashed, Line[{{1, -0.05}, {1, 1.05}}]}},
    {Orange, {Thickness[0.004], Dashed, Line[{{0.8, -0.3}, {2, -0.3}}]}},
    {Orange, {Thickness[0.004], Dashed, Line[{{1.2, -1.3}, {2, -0.3}}]}},
    {Blue, Arrowheads[0.06], {Thickness[0.006], Arrow[{{1.2, -1.3}, {0.8, -0.3}}]}},
    {Blue, Arrowheads[0.06], {Thickness[0.006], Arrow[{{0, -1.3}, {2, -0.3}}]}},
    {Blue, Arrowheads[0.06], {Thickness[0.006], Arrow[{{0, -1.3}, {1, -0.8}}]}},
    Text[Style["V", 24, FontFamily → "Euclid Math One", White], {0.45, 0.5}],
    Text[Style["A", 17, FontFamily → "Arial", White], {0.513, 0.456}],
    Text[Style["V", 24, FontFamily → "Euclid Math One", White], {1.55, 0.5}],
    Text[Style["B", 17, FontFamily → "Arial", White], {1.616, 0.455}],
    Text[Style["D", 24, FontFamily → "Arial", Orange, Italic], {0.982, 0.63}],
    Text[Style["A", 17, FontFamily → "Arial", Orange], {1.063, 0.59}],
    Text[Style["D", 24, FontFamily → "Arial", Orange, Italic], {0.982, 0.38}],
    Text[Style["B", 17, FontFamily → "Arial", Orange], {1.065, 0.34}],
    Text[Style["Φ", 24, FontFamily → "Arial", Orange], {1.06, 1.08}],
    Text[Style["O", 24, FontFamily → "Arial", Orange], {0, -1.39}],
    Text[Style["B", 24, FontFamily → "Arial", RGBColor[178/255, 252/255, 61/255, 0.5]], {1.2, -1.39}],
    Text[Style["A", 24, FontFamily → "Arial", RGBColor[178/255, 252/255, 61/255]], {0.7, -0.28}],
    Text[Style["C", 24, FontFamily → "Arial", Orange], {2.02, -0.4}],
    Text[Style["M", 24, FontFamily → "Arial", Orange], {0.973, -0.932}],
    Inset[Style["a", Black, Bold, FontFamily → "Arial", FontSize → 24], {0.034, 1.12}],
    Inset[Style["b", Black, Bold, FontFamily → "Arial", FontSize → 24], {0.034, -0.2}]}];
Export[MyDirection <> "figure3.png", cc, Background → None, ImageResolution → 1200];

## ## ## ## ## ## ## ## ## ## ## Figure3 ## ## ## ## ## ## ## ## ## ## ## ## ## ## ##

## ## ## ## ## ## ## ## ## ## ## Figure4 ## ## ## ## ## ## ## ## ## ## ## ## ## ## ##
```

```
In[ ]:= Clear["Global`*"];
text = Graphics[{Gray, Line[{{1, 0}, {1, 10}}], Line[{{2, 0}, {2, 10}}],
    Line[{{3, 0}, {3, 10}}], Line[{{4, 0}, {4, 10}}], Line[{{5, 0}, {5, 10}}],
    Line[{{6, 0}, {6, 10}}], Line[{{7, 0}, {7, 10}}], Line[{{8, 0}, {8, 10}}], Line[{{9, 0}, {9, 10}}],
    Line[{{0, 1}, {10, 1}}], Line[{{0, 2}, {10, 2}}], Line[{{0, 3}, {10, 3}}], Line[{{0, 4}, {10, 4}}],
    Line[{{0, 5}, {10, 5}}], Line[{{0, 6}, {10, 6}}], Line[{{0, 7}, {10, 7}}], Line[{{0, 8}, {10, 8}}],
    Line[{{0, 9}, {10, 9}}], Orange, Rectangle[{6, 4}, {7, 5}]}, PlotRangePadding → 1/1000];
dd = Show[{Plot3D[Sin[x + Cos[y]], {x, −3, 3}, {y, −3, 3}, PlotPoints → 60, MaxRecursion → 3,
     PlotStyle → Texture[text], Mesh → None, Lighting → "Neutral", PlotLabels → Placed["", {0, 0}],
     BoundaryStyle → None, Boxed → False, Axes → None, ViewPoint → {1, −1.9, 1.4}],
    Graphics3D[{{Thickness[0.007], Black,
       Arrow[{{0, 0, 0}, {−Evaluate[D[Sin[x + Cos[y]], x] /. {x → 0.88, y → −0.3}],
          −Evaluate[D[Sin[x + Cos[y]], y] /. {x → 0.88, y → −0.3}], 1}} +
         {{0.88, −0.3, Sin[0.88 + Cos[−0.3]]}, {0.88, −0.3, Sin[0.88 + Cos[−0.3]]}}]},
      {Text[Style["N", 14, FontFamily → "Arial", Bold, Italic, Black],
        {−Evaluate[D[Sin[x + Cos[y]], x] /. {x → 0.88, y → −0.3}],
          −Evaluate[D[Sin[x + Cos[y]], y] /. {x → 0.88, y → −0.3}], 1} +
         {0.88, −0.3, Sin[0.88 + Cos[−0.3]]} + {0.02, 0.03, 0.23}]},
      {Thickness[0.007], Blue, Arrow[{{0.88, −0.3, Sin[0.88 + Cos[−0.3]]}, {1.88, −0.5, 2}}]},
      {Text[Style["X", 14, FontFamily → "Euclid Math One", Bold, Blue], {2.01, −0.5, 2.01}]},
      {Text[Style["Σ", 14, FontFamily → "Arial", Italic, Gray], {−2.14, −1.5, 0.7}]},
      {Text[Style["dS", 14, FontFamily → "Arial", Orange], {0.55, −0.8, 1.39}]}}]}];
dd = Pane[dd, {400, 300}, ImageMargins → {{−8, −52}, {−74, −39}}];
Export[MyDirection <> "figure4.png", dd, Background → None, ImageResolution → 1200];
```

Figure4 ## ## ## ## ## ## ## ## ## ## ## ## ##

Figure5 ## ## ## ## ## ## ## ## ## ## ## ## ##

```
In[ ]:= Clear["Global`*"];
head = Graphics[Polygon[0.3 * {{−1, 8.09/25}, {0, 0}, {−1, −8.09/25}, {−8.09/10, 0}, {−1, 8.09/25}}]];
headv = Graphics[Polygon[0.3 * {{−1, 8.09/25}, {0, 0}, {−1, −8.09/25}, {−1, 8.09/25}}]];
p =
  {{RandomReal[{1.1, 1.9}], RandomReal[{3.1, 3.9}]}, {RandomReal[{5.1, 5.9}], RandomReal[{7.1, 7.9}]},
    {RandomReal[{6.1, 6.9}], RandomReal[{5.1, 5.9}]}, {RandomReal[{8.1, 8.9}], RandomReal[{5.1, 5.9}]},
    {RandomReal[{8.1, 8.9}], RandomReal[{1.1, 1.9}]}, {RandomReal[{2.1, 2.5}], RandomReal[{6.1, 6.9}]},
    {RandomReal[{2.6, 2.9}], RandomReal[{6.1, 6.9}]}, {RandomReal[{3.1, 3.5}], RandomReal[{1.1, 1.9}]},
    {RandomReal[{3.6, 3.9}], RandomReal[{1.1, 1.9}]}, {RandomReal[{3.1, 3.5}], RandomReal[{8.1, 8.9}]},
    {RandomReal[{3.6, 3.9}], RandomReal[{8.1, 8.9}]}, {RandomReal[{4.1, 4.5}], RandomReal[{4.1, 4.9}]},
    {RandomReal[{4.6, 4.9}], RandomReal[{4.1, 4.9}]}, {RandomReal[{7.1, 7.5}], RandomReal[{7.1, 7.9}]},
    {RandomReal[{7.6, 7.9}], RandomReal[{7.1, 7.9}]}, {RandomReal[{4.1, 4.3}], RandomReal[{2.1, 2.9}]},
    {RandomReal[{4.4, 4.6}], RandomReal[{2.1, 2.9}]}, {RandomReal[{4.7, 4.9}], RandomReal[{2.1, 2.9}]},
    {RandomReal[{5.1, 5.3}], RandomReal[{6.1, 6.9}]}, {RandomReal[{5.4, 5.6}], RandomReal[{6.1, 6.9}]},
    {RandomReal[{5.7, 5.9}], RandomReal[{6.1, 6.9}]}, {RandomReal[{6.1, 6.3}], RandomReal[{3.1, 3.9}]},
    {RandomReal[{6.4, 6.6}], RandomReal[{3.1, 3.9}]}, {RandomReal[{6.7, 6.9}], RandomReal[{3.1, 3.9}]},
    {RandomReal[{8.1, 8.3}], RandomReal[{3.1, 3.9}]}, {RandomReal[{8.4, 8.6}], RandomReal[{3.1, 3.9}]},
    {RandomReal[{8.7, 8.9}], RandomReal[{3.1, 3.9}]}};
ee = Graphics[{Gray, Line[{{1, 0}, {1, 10}}], Line[{{2, 0}, {2, 10}}],
    Line[{{3, 0}, {3, 10}}], Line[{{4, 0}, {4, 10}}], Line[{{5, 0}, {5, 10}}], Line[{{6, 0}, {6, 10}}],
    Line[{{7, 0}, {7, 10}}], Line[{{8, 0}, {8, 10}}], Line[{{9, 0}, {9, 10}}], Line[{{0, 1}, {10, 1}}],
    Line[{{0, 2}, {10, 2}}], Line[{{0, 3}, {10, 3}}], Line[{{0, 4}, {10, 4}}], Line[{{0, 5}, {10, 5}}],
    Line[{{0, 6}, {10, 6}}], Line[{{0, 7}, {10, 7}}], Line[{{0, 8}, {10, 8}}], Line[{{0, 9}, {10, 9}}],
    {PointSize[0.02], Red, Point[p[[1]]]}, {Arrowheads[{{0.06, 1, {head, 0.06}}}], Black,
```

$$\text{Arrow}\left[\left\{p[[1]], \text{ReplaceAll}[\theta \to \text{RandomReal}[2\,\pi]]\left[\begin{pmatrix}\cos(\theta) & -\sin(\theta)\\ \sin(\theta) & \cos(\theta)\end{pmatrix}.\left\{\frac{2}{5}, 0\right\} + p[[1]]\right]\right\}\right]\right\},$$

{PointSize[0.02], Red, Point[p[[2]]]}, {Arrowheads[{{0.06, 1, {head, 0.06}}}], Black,

$$\text{Arrow}\left[\left\{p[[2]], \text{ReplaceAll}[\theta \to \text{RandomReal}[2\,\pi]]\left[\begin{pmatrix}\cos(\theta) & -\sin(\theta)\\ \sin(\theta) & \cos(\theta)\end{pmatrix}.\left\{\frac{2}{5}, 0\right\} + p[[2]]\right]\right\}\right]\right\},$$

{PointSize[0.02], Red, Point[p[[3]]]}, {Arrowheads[{{0.06, 1, {head, 0.06}}}], Black,

$$\text{Arrow}\left[\left\{p[[3]], \text{ReplaceAll}[\theta \to \text{RandomReal}[2\,\pi]]\left[\begin{pmatrix}\cos(\theta) & -\sin(\theta)\\ \sin(\theta) & \cos(\theta)\end{pmatrix}.\left\{\frac{2}{5}, 0\right\} + p[[3]]\right]\right\}\right]\right\},$$

{PointSize[0.02], Red, Point[p[[4]]]}, {Arrowheads[{{0.06, 1, {head, 0.06}}}], Black,

$$\text{Arrow}\left[\left\{p[[4]], \text{ReplaceAll}[\theta \to \text{RandomReal}[2\,\pi]]\left[\begin{pmatrix}\cos(\theta) & -\sin(\theta)\\ \sin(\theta) & \cos(\theta)\end{pmatrix}.\left\{\frac{2}{5}, 0\right\} + p[[4]]\right]\right\}\right]\right\},$$

{PointSize[0.02], Red, Point[p[[5]]]}, {Arrowheads[{{0.06, 1, {head, 0.06}}}], Black,

$$\text{Arrow}\left[\left\{p[[5]], \text{ReplaceAll}[\theta \to \text{RandomReal}[2\,\pi]]\left[\begin{pmatrix}\cos(\theta) & -\sin(\theta)\\ \sin(\theta) & \cos(\theta)\end{pmatrix}.\left\{\frac{2}{5}, 0\right\} + p[[5]]\right]\right\}\right]\right\},$$

{PointSize[0.02], Green, Point[p[[6]]]}, {Arrowheads[{{0.06, 1, {head, 0.06}}}], Black,

$$\text{Arrow}\left[\left\{p[[6]], \text{ReplaceAll}[\theta \to \text{RandomReal}[2\,\pi]]\left[\begin{pmatrix}\cos(\theta) & -\sin(\theta)\\ \sin(\theta) & \cos(\theta)\end{pmatrix}.\left\{\frac{2}{5}, 0\right\} + p[[6]]\right]\right\}\right]\right\},$$

{PointSize[0.02], Green, Point[p[[7]]]}, {Arrowheads[{{0.06, 1, {head, 0.06}}}], Black,

$$\text{Arrow}\left[\left\{p[[7]], \text{ReplaceAll}[\theta \to \text{RandomReal}[2\,\pi]]\left[\begin{pmatrix}\cos(\theta) & -\sin(\theta)\\ \sin(\theta) & \cos(\theta)\end{pmatrix}.\left\{\frac{2}{5}, 0\right\} + p[[7]]\right]\right\}\right]\right\},$$

{PointSize[0.02], Green, Point[p[[8]]]}, {Arrowheads[{{0.06, 1, {head, 0.06}}}], Black,

$$\text{Arrow}\left[\left\{p[[8]], \text{ReplaceAll}[\theta \to \text{RandomReal}[2\,\pi]]\left[\begin{pmatrix}\cos(\theta) & -\sin(\theta)\\ \sin(\theta) & \cos(\theta)\end{pmatrix}.\left\{\frac{2}{5}, 0\right\} + p[[8]]\right]\right\}\right]\right\},$$

{PointSize[0.02], Green, Point[p[[9]]]}, {Arrowheads[{{0.06, 1, {head, 0.06}}}], Black,

$$\text{Arrow}\left[\left\{p[[9]], \text{ReplaceAll}[\theta \to \text{RandomReal}[2\,\pi]]\left[\begin{pmatrix}\cos(\theta) & -\sin(\theta)\\ \sin(\theta) & \cos(\theta)\end{pmatrix}.\left\{\frac{2}{5}, 0\right\} + p[[9]]\right]\right\}\right]\right\},$$

{PointSize[0.02], Green, Point[p[[10]]]}, {Arrowheads[{{0.06, 1, {head, 0.06}}}], Black,

$$\text{Arrow}\left[\left\{p[[10]], \text{ReplaceAll}[\theta \to \text{RandomReal}[2\,\pi]]\left[\begin{pmatrix}\cos(\theta) & -\sin(\theta)\\ \sin(\theta) & \cos(\theta)\end{pmatrix}.\left\{\frac{2}{5}, 0\right\} + p[[10]]\right]\right\}\right]\right\},$$

{PointSize[0.02], Green, Point[p[[11]]]}, {Arrowheads[{{0.06, 1, {head, 0.06}}}], Black,

$$\text{Arrow}\left[\left\{p[[11]], \text{ReplaceAll}[\theta \to \text{RandomReal}[2\,\pi]]\left[\begin{pmatrix}\cos(\theta) & -\sin(\theta)\\ \sin(\theta) & \cos(\theta)\end{pmatrix}.\left\{\frac{2}{5}, 0\right\} + p[[11]]\right]\right\}\right]\right\},$$

{PointSize[0.02], Green, Point[p[[12]]]}, {Arrowheads[{{0.06, 1, {head, 0.06}}}], Black,

$$\text{Arrow}\left[\left\{p[[12]], \text{ReplaceAll}[\theta \to \text{RandomReal}[2\,\pi]]\left[\begin{pmatrix}\cos(\theta) & -\sin(\theta)\\ \sin(\theta) & \cos(\theta)\end{pmatrix}.\left\{\frac{2}{5}, 0\right\} + p[[12]]\right]\right\}\right]\right\},$$

{PointSize[0.02], Green, Point[p[[13]]]}, {Arrowheads[{{0.06, 1, {head, 0.06}}}], Black,

$$\text{Arrow}\left[\left\{p[[13]], \text{ReplaceAll}[\theta \to \text{RandomReal}[2\,\pi]]\left[\begin{pmatrix}\cos(\theta) & -\sin(\theta)\\ \sin(\theta) & \cos(\theta)\end{pmatrix}.\left\{\frac{2}{5}, 0\right\} + p[[13]]\right]\right\}\right]\right\},$$

{PointSize[0.02], Green, Point[p[[14]]]}, {Arrowheads[{{0.06, 1, {head, 0.06}}}], Black,

$$\text{Arrow}\left[\left\{p[[14]], \text{ReplaceAll}[\theta \to \text{RandomReal}[2\,\pi]]\left[\begin{pmatrix}\cos(\theta) & -\sin(\theta)\\ \sin(\theta) & \cos(\theta)\end{pmatrix}.\left\{\frac{2}{5}, 0\right\} + p[[14]]\right]\right\}\right]\right\},$$

{PointSize[0.02], Green, Point[*p*⟦15⟧]}, {Arrowheads[{{0.06, 1, {head, 0.06}}}], Black,

Arrow[{*p*⟦15⟧, ReplaceAll[$\theta \to$ RandomReal[2π]][$\begin{pmatrix}\cos(\theta) & -\sin(\theta)\\ \sin(\theta) & \cos(\theta)\end{pmatrix}.\{\frac{2}{5}, 0\} + p⟦15⟧$]}]},

{PointSize[0.02], Blue, Point[*p*⟦16⟧]}, {Arrowheads[{{0.06, 1, {head, 0.06}}}], Black,

Arrow[{*p*⟦16⟧, ReplaceAll[$\theta \to$ RandomReal[2π]][$\begin{pmatrix}\cos(\theta) & -\sin(\theta)\\ \sin(\theta) & \cos(\theta)\end{pmatrix}.\{\frac{2}{5}, 0\} + p⟦16⟧$]}]},

{PointSize[0.02], Blue, Point[*p*⟦17⟧]}, {Arrowheads[{{0.06, 1, {head, 0.06}}}], Black,

Arrow[{*p*⟦17⟧, ReplaceAll[$\theta \to$ RandomReal[2π]][$\begin{pmatrix}\cos(\theta) & -\sin(\theta)\\ \sin(\theta) & \cos(\theta)\end{pmatrix}.\{\frac{2}{5}, 0\} + p⟦17⟧$]}]},

{PointSize[0.02], Blue, Point[*p*⟦18⟧]}, {Arrowheads[{{0.06, 1, {head, 0.06}}}], Black,

Arrow[{*p*⟦18⟧, ReplaceAll[$\theta \to$ RandomReal[2π]][$\begin{pmatrix}\cos(\theta) & -\sin(\theta)\\ \sin(\theta) & \cos(\theta)\end{pmatrix}.\{\frac{2}{5}, 0\} + p⟦18⟧$]}]},

{PointSize[0.02], Blue, Point[*p*⟦19⟧]}, {Arrowheads[{{0.06, 1, {head, 0.06}}}], Black,

Arrow[{*p*⟦19⟧, ReplaceAll[$\theta \to$ RandomReal[2π]][$\begin{pmatrix}\cos(\theta) & -\sin(\theta)\\ \sin(\theta) & \cos(\theta)\end{pmatrix}.\{\frac{2}{5}, 0\} + p⟦19⟧$]}]},

{PointSize[0.02], Blue, Point[*p*⟦20⟧]}, {Arrowheads[{{0.06, 1, {head, 0.06}}}], Black,

Arrow[{*p*⟦20⟧, ReplaceAll[$\theta \to$ RandomReal[2π]][$\begin{pmatrix}\cos(\theta) & -\sin(\theta)\\ \sin(\theta) & \cos(\theta)\end{pmatrix}.\{\frac{2}{5}, 0\} + p⟦20⟧$]}]},

{PointSize[0.02], Blue, Point[*p*⟦21⟧]}, {Arrowheads[{{0.06, 1, {head, 0.06}}}], Black,

Arrow[{*p*⟦21⟧, ReplaceAll[$\theta \to$ RandomReal[2π]][$\begin{pmatrix}\cos(\theta) & -\sin(\theta)\\ \sin(\theta) & \cos(\theta)\end{pmatrix}.\{\frac{2}{5}, 0\} + p⟦21⟧$]}]},

{PointSize[0.02], Blue, Point[*p*⟦22⟧]}, {Arrowheads[{{0.06, 1, {head, 0.06}}}], Black,

Arrow[{*p*⟦22⟧, ReplaceAll[$\theta \to$ RandomReal[2π]][$\begin{pmatrix}\cos(\theta) & -\sin(\theta)\\ \sin(\theta) & \cos(\theta)\end{pmatrix}.\{\frac{2}{5}, 0\} + p⟦22⟧$]}]},

{PointSize[0.02], Blue, Point[*p*⟦23⟧]}, {Arrowheads[{{0.06, 1, {head, 0.06}}}], Black,

Arrow[{*p*⟦23⟧, ReplaceAll[$\theta \to$ RandomReal[2π]][$\begin{pmatrix}\cos(\theta) & -\sin(\theta)\\ \sin(\theta) & \cos(\theta)\end{pmatrix}.\{\frac{2}{5}, 0\} + p⟦23⟧$]}]},

{PointSize[0.02], Blue, Point[*p*⟦24⟧]}, {Arrowheads[{{0.06, 1, {head, 0.06}}}], Black,

Arrow[{*p*⟦24⟧, ReplaceAll[$\theta \to$ RandomReal[2π]][$\begin{pmatrix}\cos(\theta) & -\sin(\theta)\\ \sin(\theta) & \cos(\theta)\end{pmatrix}.\{\frac{2}{5}, 0\} + p⟦24⟧$]}]},

{PointSize[0.02], Blue, Point[*p*⟦25⟧]}, {Arrowheads[{{0.06, 1, {head, 0.06}}}], Black,

Arrow[{*p*⟦25⟧, ReplaceAll[$\theta \to$ RandomReal[2π]][$\begin{pmatrix}\cos(\theta) & -\sin(\theta)\\ \sin(\theta) & \cos(\theta)\end{pmatrix}.\{\frac{2}{5}, 0\} + p⟦25⟧$]}]},

{PointSize[0.02], Blue, Point[*p*⟦26⟧]}, {Arrowheads[{{0.06, 1, {head, 0.06}}}], Black,

Arrow[{*p*⟦26⟧, ReplaceAll[$\theta \to$ RandomReal[2π]][$\begin{pmatrix}\cos(\theta) & -\sin(\theta)\\ \sin(\theta) & \cos(\theta)\end{pmatrix}.\{\frac{2}{5}, 0\} + p⟦26⟧$]}]},

{PointSize[0.02], Blue, Point[*p*⟦27⟧]}, {Arrowheads[{{0.06, 1, {head, 0.06}}}], Black,

Arrow[{*p*⟦27⟧, ReplaceAll[$\theta \to$ RandomReal[2π]][$\begin{pmatrix}\cos(\theta) & -\sin(\theta)\\ \sin(\theta) & \cos(\theta)\end{pmatrix}.\{\frac{2}{5}, 0\} + p⟦27⟧$]}]},

**{Arrowheads[{{0.11, 1, {headv, 0.06}}}], Gray, Thickness[0.003],
Arrow[{{5.28, −0.78}, {9.3, −0.78}}]}, {Arrowheads[{{0.11, 1, {headv, 0.06}}}],
Gray, Thickness[0.003], Arrow[{{4.72, −0.78}, {0.7, −0.78}}]},**

```
Text[Style["V", 15, FontFamily → "Euclid Math One", Gray], Scaled[{0.5, 0.01634}]]}];
Export[MyDirection <> "figure5.png", ee, Background → None, ImageResolution → 1200];
## ## ## ## ## ## ## ## ## ## ## Figure5 ## ## ## ## ## ## ## ## ## ## ## ## ## ## ##
## ## ## ## ## ## ## ## ## ## ## Figure6 ## ## ## ## ## ## ## ## ## ## ## ## ## ## ##
```

This code takes approximately 32 minutes.

```
In[∘]:= Clear["Global`*"];
usol = Block[{ϵ = $MachineEpsilon},
    NDSolveValue[{i D[M[r, t], t] == -e^(-M[r,t]) (D[M[r, t], r, r] - (D[M[r, t], r])^2 + 2 D[M[r, t], r]/r),
      M[r, 0] == 10^-2 e^(-r^2/2), M^(1,0)[ϵ, t] == 0, M[1000, t] == 0}, M, {r, ϵ, 3}, {t, 0, 3}, Method →
      {"MethodOfLines", "SpatialDiscretization" → {"TensorProductGrid", "MinPoints" → 12000}}]];
vsol =
  Block[{ϵ = $MachineEpsilon}, NDSolveValue[{i D[M[r, t], t] == 2/r D[M[r, t], {r, 1}] + D[M[r, t], {r, 2}],
      M[r, 0] == e^(-r^2/2), M^(1,0)[ϵ, t] == 0, M[1000, t] == 0}, M, {r, ϵ, 3}, {t, 0, 3}, Method →
      {"MethodOfLines", "SpatialDiscretization" → {"TensorProductGrid", "MinPoints" → 8000}}]];
G1 = Plot3D[10^2 Norm[usol[r, t]] - Norm[vsol[r, t]], {t, 0, 3}, {r, 0, 3},
    PlotPoints → 60, MaxRecursion → 3, PlotRange → {{0, 3}, {0, 3}, {-0.003, 0.0075}},
    MeshStyle → GrayLevel[0.4], BoundaryStyle → GrayLevel[0.4],
    AxesLabel → {Style["t ", Italic], Style["r", Italic], Rotate["Δρ                ", π/2]},
    AxesStyle → Directive[Black, Thickness → 0.002],
    BoxStyle → Directive[Black, Thickness → 0.0021], TicksStyle → Black,
    LabelStyle → Directive[Black, FontFamily → "Arial", FontSize → 20], ViewPoint → {1, -2, 2.1}];
FindMaxValue[{(10^2 Norm[usol[r, t]] - Norm[vsol[r, t]]), r > 0, t > 0}, {r, t}] /
  (Norm[vsol[r, t]] /. Last[FindMaximum[{10^2 Norm[usol[r, t]] - Norm[vsol[r, t]], r > 0, t > 0}, {r, t}]])
vsol = Block[{ϵ = $MachineEpsilon},
    NDSolveValue[{i D[M[r, t], t] == -e^(-M[r,t]) (D[M[r, t], r, r] - (D[M[r, t], r])^2 + 2 D[M[r, t], r]/r),
      M[r, 0] == 1/2 e^(-r^2/2), M^(1,0)[ϵ, t] == 0, M[1000, t] == 0}, M, {r, ϵ, 4}, {t, 0, 2}, Method →
      {"MethodOfLines", "SpatialDiscretization" → {"TensorProductGrid", "MinPoints" → 21000}}]];
xv = NArgMax[Norm[vsol[0, t]], {t, 0.1, 0.5}];
G2 = Show[Plot3D[2 Norm[vsol[r, t]], {r, 0, 3}, {t, 0, 2},
     PlotRange → All, MeshStyle → GrayLevel[0.4], BoundaryStyle → GrayLevel[0.4],
     AxesLabel → {Style["r ", Italic], Style["t", Italic], Rotate["ρ", π/2]},
     AxesStyle → Directive[Black, Thickness → 0.002], BoxStyle → Directive[Black, Thickness → 0.002],
     TicksStyle → Black, LabelStyle → Directive[Black, FontFamily → "Arial", FontSize → 20],
     ViewPoint → {3, -2.2, 4.1}], ParametricPlot3D[{r, xv, 2 Norm[vsol[r, xv]]},
     {r, 0, 3}, PlotStyle → Directive[Red, Thickness → 0.005]]];
usol = Block[{ϵ = $MachineEpsilon},
```

```
    NDSolveValue[{i D[M[r, t], t] == -e^(-M[r,t]) (D[M[r, t], r, r] - (D[M[r, t], r])^2 + (2 D[M[r, t], r])/r),
      M[r, 0] == 1/4 e^(-r^2/2), M^(1,0)[ϵ, t] == 0, M[1000, t] == 0}, M, {r, ϵ, 4}, {t, 0, 2}, Method →
      {"MethodOfLines", "SpatialDiscretization" → {"TensorProductGrid", "MinPoints" → 12 000}}]];
xu = NArgMax[Norm[usol[0, t]], {t, 0, 0.2}];
wsol = Block[{ϵ = $MachineEpsilon},
    NDSolveValue[{i D[M[r, t], t] == -e^(-M[r,t]) (D[M[r, t], r, r] - (D[M[r, t], r])^2 + (2 D[M[r, t], r])/r),
      M[r, 0] == 5/8 e^(-r^2/2), M^(1,0)[ϵ, t] == 0, M[1000, t] == 0}, M, {r, ϵ, 4}, {t, 0, 11/20}, Method →
      {"MethodOfLines", "SpatialDiscretization" → {"TensorProductGrid", "MinPoints" → 11 000}}]];
xw = NArgMax[Norm[wsol[0, t]], {t, 0.1, 0.5}];
xsol = Block[{ϵ = $MachineEpsilon},
    NDSolveValue[{i D[M[r, t], t] == -e^(-M[r,t]) (D[M[r, t], r, r] - (D[M[r, t], r])^2 + (2 D[M[r, t], r])/r),
      M[r, 0] == 3/4 e^(-r^2/2), M^(1,0)[ϵ, t] == 0, M[1000, t] == 0}, M, {r, ϵ, 4}, {t, 0, 11/20}, Method →
      {"MethodOfLines", "SpatialDiscretization" → {"TensorProductGrid", "MinPoints" → 12 000}}]];
xx = NArgMax[Norm[xsol[0, t]], {t, 0.1, 0.5}];
G3 = Plot[{4 Norm[usol[r, xu]], 2 Norm[vsol[r, xv]], 8/5 Norm[wsol[r, xw]], 4/3 Norm[xsol[r, xx]]},
    {r, 0, 3}, PlotRange → {{0, 3}, {-0.02, 1.42}}, PlotStyle → {{Black, Thickness → 0.005},
      {Red, Thickness → 0.005}, {Green, Thickness → 0.005}, {Blue, Thickness → 0.005}},
    Frame → {{True, False}, {True, False}}, FrameStyle → Directive[Black, Thickness → 0.002],
    FrameLabel → {Style["r", Italic], Style["ρ", Plain]},
    LabelStyle → Directive[Black, FontFamily → "Arial", FontSize → 20],
    Epilog → Inset[LineLegend[{Directive[Blue, Thickness[0.005]], Directive[Green, Thickness[0.005]],
          Directive[Red, Thickness[0.005]], Directive[Black, Thickness[0.005]]}, {Style["0.750", 20,
           FontFamily → "Arial", Blue], Style["0.625", 20, FontFamily → "Arial", Green], Style[
           "0.500", 20, FontFamily → "Arial", Red], Style["0.250", 20, FontFamily → "Arial", Black]},
        LegendFunction → (Framed[#, RoundingRadius → 5, FrameStyle → GrayLevel[0.58]] &)],
      Scaled[{0.773, 0.667}]]];

M[x_, y_, z_] := -log(((rc - re e^Mc) Sqrt[x^2 + y^2 + z^2])/(e^Mc - 1) + rc re) + log((e^Mc (rc - re))/(e^Mc - 1)) + 1/2 log(x^2 + y^2 + z^2);

rc = 1/6000;
re = 30;
Mc = 3 + i;
Ω = ImplicitRegion[rc^2 ≤ x^2 + y^2 ≤ re^2, {x, y}];
G4 = DensityPlot[
    NIntegrate[Norm[M[x, y, z]], {z, -Sqrt[re^2 - x^2 - y^2], Sqrt[re^2 - x^2 - y^2]}, MaxRecursion → 15], {x, y} ∈ Ω,
    PlotRange → {{-30.07, 30.07}, {-30.07, 30.07}, {0, Sqrt[10]}}, ColorFunction → (Hue[0.65, #1] &),
    Frame → False, PlotPoints → 1000, Epilog → {Directive[Thickness[0.0014], Gray], Circle[{0, 0}, 30]}];
```

$$\mathcal{M}[\mathrm{r\_}] := -\log\left(\frac{r\left(\mathrm{rc}-\mathrm{re}\,e^{\mathcal{M}\mathrm{c}}\right)}{\mathrm{rc}\,\mathrm{re}\left(e^{\mathcal{M}\mathrm{c}}-1\right)}+1\right)+\log(r)+\log\left(\frac{e^{\mathcal{M}\mathrm{c}}\left(\mathrm{rc}-\mathrm{re}\right)}{\mathrm{rc}\left(e^{\mathcal{M}\mathrm{c}}-1\right)}\right)-\log(\mathrm{re});$$

$$\mathcal{M}\mathrm{c} = 3 + i;$$

$$\mathrm{rc} = \frac{1}{6000};$$

$$\mathrm{re} = 30;$$

$$A = \frac{1}{26\,300};$$

$$B = \frac{22}{5};$$

$$\mathrm{G5} = \mathrm{LogLogPlot}\left[\left\{\mathrm{Norm}[\mathcal{M}[r]],\ \frac{A}{\frac{r}{B}\left(1+\frac{r}{B}\right)^2}\right\},\ \left\{r,\ \frac{1}{6000},\ 3\right\},\ \mathrm{PlotRange} \to \{\{0, 3\}, \{0, 3\}\},\right.$$

```
    PlotStyle → {Directive[Orange, Thickness[0.005]], Directive[Green, Dashed, Thickness[0.005]]},
    Frame → {{True, False}, {True, False}}, FrameLabel → {Style["r", Italic], " ρ"},
    FrameStyle → Directive[Black, Thickness → 0.0021],
    LabelStyle → Directive[Black, FontFamily → "Arial", FontSize → 20],
    Epilog → Inset[LineLegend[{Directive[Orange, Thickness[0.004]], Directive[Green, Thickness[0.004]]},
        {Style["this study", FontFamily → "Arial", FontSize → 20],
          Style["NFW", FontFamily → "Arial", FontSize → 20]}, LegendFunction →
          (Framed[#, RoundingRadius → 4, FrameStyle → GrayLevel[0.58]] &)], Scaled[{0.73, 0.74}]]];
```

$$\mathrm{G6} = \mathrm{LogLogPlot}\left[\left\{4\,\mathrm{Norm}[\mathrm{usol}[r, \mathrm{xu}]],\ 2\,\mathrm{Norm}[\mathrm{vsol}[r, \mathrm{xv}]],\ \frac{8}{5}\,\mathrm{Norm}[\mathrm{wsol}[r, \mathrm{xw}]],\ \frac{4}{3}\,\mathrm{Norm}[\mathrm{xsol}[r, \mathrm{xx}]]\right\},\right.$$

```
    {r, 0, 4}, PlotRange → All, PlotStyle → {{Black, Thickness → 0.005}, {Red, Thickness → 0.005},
      {Green, Thickness → 0.005}, {Blue, Thickness → 0.005}}, Frame → {{True, False}, {True, False}},
    FrameStyle → Directive[Black, Thickness → 0.002], FrameLabel → {Style["r", Italic], Style["ρ", Plain]},
    LabelStyle → Directive[Black, FontFamily → "Arial", FontSize → 20],
    FrameTicks → {{{0.004, "", {0.007, 0}, Thickness → 0.0017}, {0.005, "", {0.007, 0}, Thickness → 0.0017},
        {0.006, "", {0.007, 0}, Thickness → 0.0017}, {0.007, "", {0.007, 0}, Thickness → 0.0017},
        {0.008, "", {0.007, 0}, Thickness → 0.0017}, {0.009, "", {0.007, 0}, Thickness → 0.0017},
        {0.01, "0.01", {0.01, 0}, Thickness → 0.0017}, {0.02, "", {0.007, 0}, Thickness → 0.0017},
        {0.03, "", {0.007, 0}, Thickness → 0.0017}, {0.04, "", {0.007, 0}, Thickness → 0.0017},
        {0.05, "", {0.007, 0}, Thickness → 0.0017}, {0.06, "", {0.007, 0}, Thickness → 0.0017},
        {0.07, "", {0.007, 0}, Thickness → 0.0017}, {0.08, "", {0.007, 0}, Thickness → 0.0017},
        {0.09, "", {0.007, 0}, Thickness → 0.0017}, {0.1, "0.1", {0.01, 0}, Thickness → 0.0017},
        {0.2, "", {0.007, 0}, Thickness → 0.0017}, {0.3, "", {0.007, 0}, Thickness → 0.0017},
        {0.4, "", {0.007, 0}, Thickness → 0.0017}, {0.5, "", {0.007, 0}, Thickness → 0.0017},
        {0.6, "", {0.007, 0}, Thickness → 0.0017}, {0.7, "", {0.007, 0}, Thickness → 0.0017},
        {0.8, "", {0.007, 0}, Thickness → 0.0017}, {0.9, "", {0.007, 0}, Thickness → 0.0017},
        {1, "1", {0.01, 0}, Thickness → 0.0017}, {2, "", {0.007, 0}, Thickness → 0.0017},
        {3, "", {0.007, 0}, Thickness → 0.0017}, {4, "", {0.007, 0}, Thickness → 0.0017}},
      {{0.0001, "", {0.007, 0}, Thickness → 0.0017}, {0.0002, "", {0.007, 0}, Thickness → 0.0017},
        {0.0003, "", {0.007, 0}, Thickness → 0.0017}, {0.0004, "", {0.007, 0}, Thickness → 0.0017},
        {0.0005, "", {0.007, 0}, Thickness → 0.0017}, {0.0006, "", {0.007, 0}, Thickness → 0.0017},
        {0.0007, "", {0.007, 0}, Thickness → 0.0017}, {0.0008, "", {0.007, 0}, Thickness → 0.0017},
        {0.0009, "", {0.007, 0}, Thickness → 0.0017}, {0.001, "0.001", {0.01, 0}, Thickness → 0.0017},
        {0.002, "", {0.007, 0}, Thickness → 0.0017}, {0.003, "", {0.007, 0}, Thickness → 0.0017},
        {0.004, "", {0.007, 0}, Thickness → 0.0017}, {0.005, "", {0.007, 0}, Thickness → 0.0017},
        {0.006, "", {0.007, 0}, Thickness → 0.0017}, {0.007, "", {0.007, 0}, Thickness → 0.0017},
        {0.008, "", {0.007, 0}, Thickness → 0.0017}, {0.009, "", {0.007, 0}, Thickness → 0.0017},
        {0.01, "0.01", {0.01, 0}, Thickness → 0.0017}, {0.02, "", {0.007, 0}, Thickness → 0.0017},
        {0.03, "", {0.007, 0}, Thickness → 0.0017}, {0.04, "", {0.007, 0}, Thickness → 0.0017},
        {0.05, "", {0.007, 0}, Thickness → 0.0017}, {0.06, "", {0.007, 0}, Thickness → 0.0017},
        {0.07, "", {0.007, 0}, Thickness → 0.0017}, {0.08, "", {0.007, 0}, Thickness → 0.0017},
```

```
        {0.09, "", {0.007, 0}, Thickness → 0.0017}, {0.1, "0.1", {0.01, 0}, Thickness → 0.0017},
        {0.2, "", {0.007, 0}, Thickness → 0.0017}, {0.3, "", {0.007, 0}, Thickness → 0.0017},
        {0.4, "", {0.007, 0}, Thickness → 0.0017}, {0.5, "", {0.007, 0}, Thickness → 0.0017},
        {0.6, "", {0.007, 0}, Thickness → 0.0017}, {0.7, "", {0.007, 0}, Thickness → 0.0017},
        {0.8, "", {0.007, 0}, Thickness → 0.0017}, {0.9, "", {0.007, 0}, Thickness → 0.0017},
        {1, "1", {0.01, 0}, Thickness → 0.0017}, {2, "", {0.007, 0}, Thickness → 0.0017}}}];
ff = GraphicsGrid[{{G1, G2}, {G3, G4}, {G5, G6}}, Spacings → {10, 10}, ImageSize → 800,
   Epilog → {Text[Style["a", 21, FontFamily → "Arial", Black, Bold], Scaled[{−0.41, 1.2}]],
      Text[Style["b", 21, FontFamily → "Arial", Black, Bold], Scaled[{0.6, 1.2}]],
      Text[Style["c", 21, FontFamily → "Arial", Black, Bold], Scaled[{−0.41, 0.72}]],
      Text[Style["d", 21, FontFamily → "Arial", Black, Bold], Scaled[{0.6, 0.72}]],
      Text[Style["e", 21, FontFamily → "Arial", Black, Bold], Scaled[{−0.41, 0.212}]],
      Text[Style["f", 21, FontFamily → "Arial", Black, Bold], Scaled[{0.6, 0.212}]]}];
Export[MyDirection <> "figure6.png", ff, Background → None];
```

Out[ ]=

0.00362328

## ## ## ## ## ## ## ## ## ## ## Figure6 ## ## ## ## ## ## ## ## ## ## ## ## ## ## ##
